# Origin and evolution of papillomavirus (onco)genes and genomes

**DOI:** 10.1098/rstb.2018.0303

**Published:** 2019-04-08

**Authors:** Anouk Willemsen, Ignacio G. Bravo

**Affiliations:** Centre National de la Recherche Scientifique (CNRS), Laboratory MIVEGEC (CNRS IRD Uni Montpellier), 34090 Montpellier, France

**Keywords:** oncogenes, virus evolution, papillomaviruses, genome evolution, phylogenetic dating

## Abstract

Papillomaviruses (PVs) are ancient viruses infecting vertebrates, from fishes to mammals. Although the genomes of PVs are small and show conserved synteny, PVs display large genotypic diversity and ample variation in the phenotypic presentation of the infection. Most PV genomes contain two small early genes *E6* and *E7*. In a bunch of closely related human papillomaviruses (HPVs), the E6 and E7 proteins provide the viruses with oncogenic potential. The recent discoveries of PVs without *E6* and *E7* in different fish species place a new root on the PV tree, and suggest that ancestral PVs consisted of the minimal PV backbone *E1-E2-L2-L1*. Bayesian phylogenetic analyses date the most recent common ancestor of the PV backbone to 424 million years ago (Ma). Common ancestry tests on extant *E6* and *E7* genes indicate that they share a common ancestor dating back to at least 184 Ma. In *AlphaPVs* infecting Old World monkeys and apes, the appearance of the *E5* oncogene 53–58 Ma concurred with (i) a significant increase in substitution rate, (ii) a basal radiation and (iii) key gain of functions in E6 and E7. This series of events was instrumental to construct the extant phenotype of oncogenic HPVs. Our results assemble the current knowledge on PV diversity and present an ancient evolutionary timeline punctuated by evolutionary innovations in the history of this successful viral family.

This article is part of the theme issue ‘Silent cancer agents: multi-disciplinary modelling of human DNA oncoviruses’.

## 1. Background

Papillomaviruses (PVs) present a small, circular double-stranded DNA genome with an average size of 8 kbp. A canonical PV genome is organized into three major regions: an upstream regulatory region (URR), an early gene region encoding for three to six proteins (E6, E7, E5, E1, E2 and E4, nested in E2), and a late gene region encoding for two capsid proteins (L2 and L1) [1]. Proteins in the early region are (among other functions) involved in viral replication and cell transformation, while the capsid proteins self assemble to yield virions and encapsidate the genome. Some of the early genes are dispensable, and the minimal PV genome may contain an URR together with the *E1-E2-L2-L1* genes.

Most PVs are part and parcel of a healthy skin microbiota causing asymptomatic infections in skin and mucosa. In humans, the best studied host, certain human papillomaviruses (HPVs) may cause benign lesions, such as skin and genital warts, where the transmission of genital warts occurs primarily via sexual activity [2]. Only a limited number of evolutionary related HPVs are associated with malignant lesions, which can develop into cancer [3,4]. Oncogenic HPVs are a major public health concern as they are responsible for virtually all cases of cervical (99%) and most cases of anal cancer (88%), as well as for a fraction of cancers on the vagina (78%), penis (51%), oropharynx (13–60%, depending on the geographical region) and vulva (15–48%, depending on age) [5]. In oncogenic HPVs, the E5, E6 and E7 proteins are directly involved in the onset of cancer. Specific cellular activities of these genes are linked to the virus oncogenic potential and PVs that do not contain these genes are not associated with cancer. In oncogenic PVs, E6 is able to induce degradation of the p53 tumour suppressor protein [6–8], while the E7 protein degrades members of the retinoblastoma protein (pRb) family [9], which also act as tumour suppressors. The E5 protein in oncogenic PVs is involved in evasion of the immune response and decreases the cellular dependence on external growth factors, thus inducing cell proliferation [10].

PVs were first isolated in mammals, but were later also found to infect birds, turtles, snakes and fish. The first fish PV was recently discovered in skin lesions in a bony fish and named after its host *Sparus aurata papillomavirus 1* (SaurPV1) [11]. The SaurPV1 genome exhibits a unique organization—to date, it is the only PV that consists of the minimal PV backbone. Moreover, the nucleotide sequences of SaurPV1 are so divergent that its discovery led to the recent proposal of reorganizing the *Papillomaviridae* taxonomy into two subfamilies: *Firstpapillomavirinae*, containing 52 genera, and *Secondpapillomavirinae*, containing one new genus to which SaurPV1 belongs.

PVs have evolved in close relationship with their hosts, which allows phylogenetic inference based on host fossil records. However, PV diversity cannot be explained by virus–host codivergence alone. As shown in previous studies [12,13], distantly related PVs infect the same host species, suggesting independent codivergence between viruses within clades and their hosts. The growing number of animal PV sequences available in the online databases allow us to add pieces to the puzzle on the origin and evolution of PV genes and genomes. The modular structure of the PV genome and the genome organization of PVs infecting fish reinforce the proposed evolutionary scenario of an ancestral PV that did not contain any of the *E5*, *E6* or *E7* genes, which would have been subsequently acquired during PV evolution [14].

To better understand how certain HPVs became oncogenic, we have set up a global dating study to look into the evolutionary history of the PV genes and genomes. We have payed special attention to the origin of the PV oncogenes, assessing whether they are monophyletic or have instead originated in several convergent acquisition/loss events. We further look into the roles of these genes and how these novel functions relate to the emergence of oncogenic potential. This paper, combining novel data with the previous literature, aims to provide insight into the evolution of PVs on a dated scale.

## 2. Material and methods

### (a) Data collection and alignments

We downloaded 354 full-length PV genome sequences (154 animal and 200 human) from the PaVE (pave.niaid.nih.gov, [15]) and GenBank (https://www.ncbi.nlm.nih.gov/genbank/) databases (electronic supplementary material, table S1). The previously identified recombinant PVs isolated from cetaceans (PphoPV1-2, TtruPV1-7, DdelPV1, PspiPV1) [16–18] were removed from the dataset, leaving us with a dataset of 343 full length PV genomes. The *E6*, *E7*, *E1*, *E2*, *E5*, *L2* and *L1* genes were extracted and aligned individually at the amino acid level using MAFFT v.7.271 [19], corrected manually and backtranslated to nucleotides using PAL2NAL v.14 [20]. The alignment was filtered using Gblocks v.0.91b [21], such that uninformative positions were removed. For *E1*, *E2*, *L2* and *L1* the original alignments contained 3516, 4014, 5070 and 2610 nucleotide positions, respectively. After Gblocks filtering these alignments contained 1665, 864, 933 and 1398 positions, respectively. For tree construction, the *E1*, *E2*, *L2* and *L1* were concatenated using a custom perl script. The *E6*, *E7* and *E5* oncogenes were tested for presence/absence in the dataset. The size of these genes was calculated for each PV, and in some cases, the annotation was manually corrected before alignment.

### (b) Phylogenetic analyses and dating

For the concatenated *E1-E2-L2-L1* alignment a maximum-likelihood (ML) tree was constructed at the nucleotide level, using RAxML v.8.2.9, under the GTR+*Γ*4 model, using 12 partitions (three for each gene, corresponding to each codon position) and 1000 bootstrap replicates (electronic supplementary material, figure S1A). The tree was rooted using the SaurPV1 sequence. Based on the best-sampled ML tree, 18 calibration points were selected on subtrees where the *E1-E2* and *L2-L1* trees did not show discrepancies and where the host tree matched the PV tree. Calibration point estimates were based on host fossil records from TimeTree (http://www.timetree.org/). The effect of the calibration points, and therewith forced clades, on the topology of the tree was validated by constructing an ML tree constrained to the calibrations used (electronic supplementary material, figure S1B) and subsequent comparison to the unconstrained tree using a Shimodaira–Hasegawa test [22], as implemented in RAxML. The constrained tree (likelihood: −977756.687001) was not significantly worse than the unconstrained tree (likelihood: −977683.344298) at the 2% significance level (ΔLH: −73.342703; s.d.: 32.00218).

Bayesian time inference was performed at the nucleotide level using BEAST v.1.8.3 [23], under the GTR+*Γ*4 model, using 12 partitions, the uncorrelated relaxed clock model [24] with a lognormal distribution and a continuous quantile parameterization [25] and the Yule Speciation Process tree prior [26,27]. The constrained ML tree was used as starting tree for time inference. Two independent MCMC chains were run for a maximum of 10^9^ generations and combined when convergence was reached. Chain #1 consisted of 5.249 × 10^8^ states with an effective sample size (ESS) of 1464, 419 and 4169 for the posterior, prior and likelihood, respectively. Chain #2 consisted of 5.265 × 10^8^ states with an ESS of 1227, 360 and 3110 for the posterior, prior and likelihood, respectively. An uncollapsed version of the tree figure with the two chains combined can be found in electronic supplementary material, figure S2.

### (c) Common ancestry test for the *E6* and *E7* oncogenes

**Figure 1.**
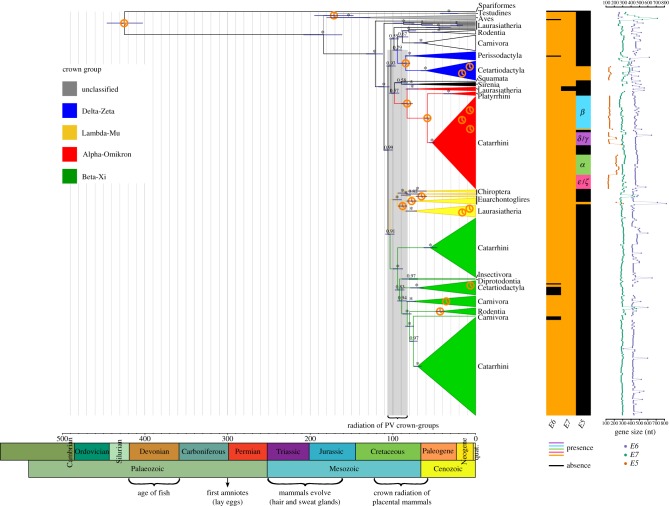
Dated Bayesian phylogenetic tree for a dataset containing 343 PVs. The tree was constructed at the nucleotide level based on the concatenated *E1-E2-L2-L1* genes. The scale bar is given in million years ago (Ma). Values at the nodes correspond to posterior probabilities, where asterisks indicate full support. Error bars encompass 95% highest posterior density for the age of the nodes. Clock symbols indicate the nodes used for calibration. Clades are coloured according to the PV crown group classification, as indicated in the legend on the left. Next to the tree on the right, the taxonomic group (superorder, class, order, parvorder, no rank) corresponds to the one in which the host clades could best be summarized. Below the tree, a geological time scale is drawn. The matrix next to the taxonomic host groups indicates the presence/absence of the *E6*, *E7* and *E5* genes for each PV (see legend), and the classification of E5 (*α*, *β*, *γ*, *δ*, *ε*, *ζ*) is indicated within the matrix. Next to the matrix, the size of the oncogenes is plotted. (Online version in colour.)

To test whether the extant *E6* and *E7* genes have a single common ancestor, we used the software Bali-Phy [28]. In this approach, the input data are the unaligned sequences, where the alignment is one of the parameters to be treated as an unknown random variable [29]. We ran our analysis on two different reduced datasets (indicated in electronic supplementary material, table S1), containing representative species from the different PV clades in figure 1. Each reduced dataset contained 35 sequences representing the different PV crown groups, including the seven PVs infecting Aves and Testudines (grey clade), six Alpha-Omikron PVs (red clade), six Beta–Xi PVs (green clade), six Lambda–Mu PVs (yellow clade), six Delta–Zeta PVs (blue clade) and the four PVs infecting manatees (black clade). For each dataset, we ran the analysis for *E6* and *E7*, both separately and concatenated. We assumed the null hypothesis (H0) of Common Ancestry (CA). The Bali-Phy analyses were performed at the amino acid level using the LG substitution model. The likelihood for the CA model was obtained running the software for all the *E6* and/or *E7* sequences together. For the different Independent Origin (IO) models, we ran the analysis for each group independently. For each IO model (H1–H7), the likelihood is represented by the sum of the likelihoods obtained for the different groups within that model. We only considered IO scenarios that were biologically plausible based on the PV tree (figure 1). For each model, three independent MCMC chains were run for 100 000 iterations. The three runs were combined and checked for convergence. Subsequently, the marginal likelihood was calculated over the three runs using the stabilized harmonic mean estimator. The Bayes factor (BF) for CA is then ΔBF = log[Prob(CA)] − log[Prob(IO)], such that positive values favour CA and negative values indicate IO. As a control, we conducted the same analyses on the *E1* genes, which are monophyletic (electronic supplementary material, table S2).

To support the results of the Bali-Phy analyses, we performed a random permutation test as described in de Oliveira Martins & Posada [30]. In this test, the sequences for one of the groups are randomly shuffled and statistics are recalculated after realignment with MUSCLE [31], which tells us how much the original data depart from those with phylogenetic structure partially removed. The statistics used in this test are ML tree lengths and log likelihoods, calculated with PhyMLv3.0 [32]. We reshuffled one of the groups 100 times, each time realigning against the other groups in the dataset. For each iteration, the alignment is always optimized and to make the statistics comparable, the same alignment is used for both the IO and CA hypotheses.

### (d) Statistics and graphics

Statistical analyses and graphics were done using R [33], with the aid of the packages ‘ape’, ‘car’, ‘ggplot2’, ‘ggtree’, ‘stats’, ‘strap’, ‘pgirmess’ and ‘reshape2’. The final display of the graphics was designed using Inkscape v.0.92 (https://inkscape.org/en/).

## 3. Results and discussion

### (a) Papillomavirus evolution: old ancestry, primary radiation and secondary diversification

Although the number of animal PV sequences in databases is growing, the number of available non-mammalian sequences remains low. At the start of this study, there were five avian, two turtle, one python and one fish PV genome sequences available in GenBank. This fish PV was recovered from lesions in the bony fish gilthead seabream (*Sparus aurata*) (SaurPV1) [11]. The genome of SaurPV1 is significantly smaller (5748 kbp) than most previously described PVs (around 8 kbp), and is unique as it is the only PV that contains the minimal PV backbone *E1-E2-L2-L1* while lacking any of the oncogenes (*E5*, *E6* and *E7*). This particular genome structure of PVs infecting fish has been confirmed by metagenomic assembly of PVs in other fish host species enriched for circular DNA viruses (GenBank accessions: MH510267, MH616908, MH617143, MH617579), which were made available during redaction of this manuscript. Besides the unique genome organization of these PVs, the distant sequence relatedness to other PVs suggests a new root on the tree (Spariformes in figure 1). A recent study dated this root to 481 Ma, albeit with a broad highest posterior density (HPD) interval (95% HPD: 326–656) [34]. In our study, the root of the tree was dated back to 424 Ma (95% HPD: 402–446) (figure 1), which is just before the Devonian period (also often named ‘Age of Fish’). These results indicate that PVs have an old ancestry: the ancestral anamniotes were already infected by ancestral PVs in the Palaeozoic, and more specifically in the Silurian period, when bony fish appeared. Millions of years later, in the Mesozoic, we find the last common ancestor of PVs infecting amniotes at around 184 Ma (95% HPD: 161–208), followed by a split of PVs infecting birds (Aves) and turtles (Testudines) (grey clade in figure 1) and PVs infecting mammals (all other PVs with one exception, described below).

The period between the last common ancestor of amniotes and the last common ancestor of mammals corresponds to the evolution of the main traits and skin structures exclusive to mammals, namely hairs, sweat glands, sebaceous glands and milk glands. It has been proposed that the modifications in the proto-mammalian skin environment increased the availability of novel cellular targets for PVs [1], so that adaptation to these new niches led to a primary radiation within PVs. A secondary diversification of PVs infecting mammals started around 120 Ma, and the radiation that generated the different PV crown groups dates back to between 106 Ma and 83 Ma, which fits well within the time of crown radiation of placental mammals (figure 1) [35]. Most PVs infecting mammals have been classified into four different crown groups: Delta–Zeta, Lambda–Mu, Alpha-Omikron and Beta–Xi (figure 1). PV crown groups are named after the two most distantly related species therein enclosed [13]. The inferred age for the ancestors of the different PV crown groups is significantly younger than the root age, and significantly different between groups (table 1). This secondary diversification event corresponds to the independent co-divergence between viruses and their hosts within each of the major viruses–host clades, generating the enormous diversity of PVs we observe today. Most of these PVs are associated with asymptomatic infections, while some of them cause productive infections, and only a few display carcinogenic potential.

**Table 1.** Inferred node age in million years ago (Ma) for the most recent common ancestors (MRCA) of the different PV clades and for the root of the tree. The rows of the PV crown groups are named accordingly (figure 1), otherwise the taxonomic host group is given. An asterisk indicates the presence of one exception within the clade of PVs infecting mammals, which is a python PV (discussed in the text). The differences between the ancestral node ages of the crown groups as well as the root are significant after performing a Kruskal–Wallis rank sum test (chi-sq. = 51993, *d.f.* = 5, *p* < 2.2 × 10^−16^) and a multiple comparison test after Kruskal–Wallis (electronic supplementary material, table S3). Although the inferred times and the posterior distributions for the ancestral Alpha–Omikron and Delta–Zeta as well as the Beta–Xi and Lambda–Mu clades are similar (electronic supplementary material, figure S3), the significant difference between these groups was confirmed by a Wilcoxon rank sum test (*W* = 57 451 000, *p* < 2.2 × 10 − 16 and *W* = 50 158 000, *p* < 2.2 × 10^16^, respectively).

PV clade	MRCA age (Ma)	95% HPD
root	424	402–446
amniotes	184	161–208
Aves/Testudines (grey)	171	148–195
mammals*	121	112–132
Lambda–Mu (yellow)	95	90–100
Beta–Xi (green)	94	88–100
Delta–Zeta (blue)	84	79–90
Alpha–Omikron (red)	83	76–90
Sirenia (black)	77	68–87

### (b) Inconsistencies of the current scenario of papillomaviruses evolution

The proposed scenario of a biphasic evolution of PVs, that is to say a primary radiation event followed by a secondary diversification, fits well globally with prior phylogenetic analyses [1,13,14], as well as with those that we present here. Nevertheless, we observe a number of flaws and anomalies in the PV trees that cannot be explained with our current, linear understanding of PV evolution. Most often, inconsistencies can be imputed to the non-systematic viral sampling, hitherto largely based on economic or leisure interests of the host species, as well as on opportunistic sampling by a reduced circle of wildlife disease scientists. Generally, a comprehensive understanding of PV evolution needs to integrate additional evolutionary mechanisms, such as lineage sorting and host switch [13], which may radically change the relationship between a viral lineage and its host species.

The first anomaly is weak consistency in time inference in the deep nodes of the PV tree, as the inferred age of the MRCA of PVs infecting amniotes (184 Ma, table 1) is more recent than the estimated divergence time between Mammalia and Sauropsida–the two main amniote lines–, which is around 312 Ma (CI: 297–326; estimate derived from 22 studies at http://www.timetree.org/). We did not force any timing interval for this node in our calibrations, so this result of a ‘younger-than-expected’ node most likely reflects the lack of sufficient phylogenetic information around it, with a long branch connecting it to the root. We will need to increase our sample size for mainly PVs infecting sauropsids and bony fish to obtain a more accurate time estimate for the MRCA of PVs infecting amniotes.

The second, conspicuous, anomaly is the presence of a PV infecting a carpet python, *Morelia spilota papillomavirus 1* (MspiPV1), well nested within mammalian PVs (Squamata in figure 1). The viral genome was retrieved from histologically determined papilloma-like neoplasias in one python, that tested negative for herpesvirus infections [36]. This PV infecting squamates is not a lone exception, as during the writing of this manuscript, the genome of a *Boa constrictor papillomavirus* (BconPV1) was made available (GenBank accession: MH605022). A standard protein BLAST [37] identified E1 and L1 of the boa PV as the best hits to the python PV with 58% and 69% of identity, respectively. We have further confirmed that the boa and python PVs are indeed sister taxa in a newly built *E1-E2-L2-L1* tree (electronic supplementary material, figure S4). This clustering supports the phylogenetic position of these two PVs infecting squamates to be close to the unresolved crown of mammalian PV crown groups. The lineages of boas and pythons split only 74 Ma (http://www.timetree.org/) and if this timing applies to the corresponding viruses, their MRCA would also fit well in the secondary diversification of PVs. Overall histological assessment of MspiPV1 in papillomatous lesions and mostly the finding of BconPV1 strongly suggest that there exists a genuine group of PVs infecting squamates that clusters with mammalian PVs rather than with PVs infecting birds and turtles. We propose that this lineage emerged after a host switch of an ancestral mammalian PV. Most likely, the evolutionary changes during the secondary PV radiation may have facilitated colonization of this new ancestral squamate host.

The third anomaly of a solitary PV branching off inconsistently with host phylogeny is a PV genome isolated from a brush-tailed bettong (*Bettongia penicillata papillomavirus 1*; BpenPV1), a rare marsupial [38]. This is the only full-genome report of PVs in marsupials, although fragmentary evidence for PVs infecting marsupials and monotremes has been communicated [39]. We report here BpenPV1 (Diprotodontia in figure 1) to be well nested within the Beta–Xi crown group, sharing a common ancestor 80 Ma (95% HPD: 70–89) with a European hedgehog PV (EeurPV1; host Insectivora in figure 1), also solitary in this crown group. Since all other PVs in this crown group have been retrieved from Laurasiatheria, we interpret that a host switch from a PV infecting placental mammals towards marsupials occurred after the emergence of the Beta–Xi crown group, generating this lineage. A systematic screening for PVs in marsupials and monotremes is seriously needed, aiming to populate the viral tree in this important clade of non-placental mammal hosts.

Finally, the fourth anomaly in the PV tree is the poor resolution around the crown of mammalian PVs. Should the null hypothesis of virus–host co-diversification be true, for placental mammals one would expect PVs infecting Xenarthra and Afrotheria to be basal to PVs infecting Euarchontoglires and Laurasiatheria. However, no PV infecting Xenarthrans has been so far identified, and within Afrotherians only a few PVs infecting manatees (black, Sirenia clade in figure 1) have been reported. These manatee PVs are clearly monophyletic, with an ancestor dating back to 77 Ma (95% HPD: 68–87). Although their position within the crown of PVs infecting placental mammals is not well resolved, they are definitely not basal to all PVs infecting Laurasiatherians and Euarchontoglires. Indeed, the MRCA of PVs infecting placental mammals dates back to 121 Ma (95% HPD: 112–132), and the most basal PV is consistently a fruit bat PV (*Rosettus aegyptiacus PV1*, RaegPV1) [40]. This virus does not cluster with any other bat PV included in this study, which actually are rather dispersed over the tree [41,42]. Once again, we interpret that the lack of appropriate host sampling prevents resolution of the mammalian PV crown.

### (c) The extant *E6* and *E7* oncogenes have a common ancestor, but extant *E5* oncogenes are not monophyletic

We have recently shown that extant *E5* oncogenes do not have a common ancestor [43]. This is not surprising as E5 proteins are only present in a few PV clades (figure 1), and are highly divergent. On the contrary, both the *E6* and *E7* oncogenes are present in most PV genomes, and are less divergent than *E5* oncogenes [3]. Interestingly, some PVs lack either *E6* or *E7* (the presence/absence matrix in figure 1). A group of PVs lacking *E7* (MricPV1, PphoPV4, SscrPV1, UmarPV1) infect laurasiatherian hosts and belong to the Alpha–Omikron PV crown group. All other members of this crown group infect Primates and present both *E6* and *E7*. On the other hand, PVs lacking *E6* are more disperse along the tree and belong to different crown groups: one parrot (PeriPV1) PV in the grey clade, one donkey PV (EasiPV1) in the Delta–Zeta crown group, and eight bovine PVs (BPV3, BPV4, BPV6, BPV9, BPV10, BPV11, BPV12, BPV15) and three HPVs (HPV101, HPV103, HPV108) in the Beta–Xi crown group.

Regarding the size of the oncogenes (indicated next to the tree in figure 1), *E6* is small (median: 253.5 nt; Q^1^: 249.0 – Q^3^: 258.0) in the genomes of PVs infecting birds and turtles (grey clade), while it is of double size (median: 438.0 nt; Q^1^: 420.0 – Q^3^: 462.0) in the genomes of all PVs infecting mammals. This increase in size correlates with the presence of a second *E6* zinc-binding motif domain [44], which could have appeared after duplication of the first original motif and transformed the original homodimer into an internal dimer [45].

As described above, during the production of this manuscript four novel fish PV genomes became available at the GenBank database, infecting three new bony fish species (one in the rainbow trout, one in the red snapper and two in the haddock). None of the genomes of these novel fish PVs contains any of the oncogenes. For the now five fish PVs, the most conserved *E1* and *L1* genes present a high sequence diversity. To see whether the novel fish PVs cluster together with the ancestral SaurPV1, we recalculated our ML tree based on the concatenated *E1-E2-L2-L1* sequences. Indeed, we found that all fish PVs are monophyletic (electronic supplementary material, figure S4).

The pattern of presence/absence of *E6* and *E7* in extant PVs demands an evolutionary explanation. One scenario would propose that the MRCA of all PVs already contained these ORFs and invokes six independent repeated loss events for *E6* in different PV lineages [46], including in the lineage leading to extant fish PVs, and one gene loss event for *E7* in another PV lineage. An alternative scenario, more parsimonious with the absence of *E6* and *E7* in fish PVs, would postulate an ancestral PV genome spanning only the minimal arrangement *E1-E2-L2-L1*, the gain of the ancestral *E6* and *E7* genes in the lineage of amniote PVs, at least 184 Ma, followed by five independent loss events for *E6* and one loss event for *E7*.

To unravel a piece of the evolutionary history of *E6* and *E7*, we performed CA tests as described in de Oliveira Martins & Posada [29] using the program Bali-Phy [28]. The power of this approach is that the alignment and the phylogeny are estimated at the same time, reducing the bias towards supporting CA introduced by the alignment step. We ran our analyses on two reduced datasets containing representatives from each PV clade. We tested different hypotheses supporting either CA or IO for *E6* and *E7* separately as well as concatenated. We only considered IO scenarios that were biologically plausible based on the PV tree, leading us to eight different hypotheses (H0–H7), as displayed in tables 2 and 3. We assumed CA as the null hypothesis (H0; Material and methods). For the IO scenarios (H1–H7), we performed the analysis separately for each group, where the sum of the marginal likelihood of these groups represents that of the hypothesis tested. As an example, for H2, we ran one analysis for the Aves/Testudines clade (grey), one analysis for the Delta–Zeta crown group (blue clade), and one analysis for the Lambda–Mu crown group (yellow clade), Alpha–Omikron crown group (red clade), Sirenia clade (black) and Beta–Xi crown group (green clade) together. Therefore, we obtained three log marginal likelihood estimates, and the sum of these rendered the likelihood for H2. Then we calculated the BF as ΔBF = log [Prob(CA)] − log [Prob(IO)], such that positive values favour CA and negative values indicate IO. The overall results suggest (tables 2 and 3) that extant *E6* and *E7* share a common ancestor, independently of whether the analyses were performed on the concatenated *E6-E7*, or on *E6* and *E7* alone. Although convergence was reached between the three independent MCMC runs for all groups within the hypotheses tested, we performed an additional permutation test to further validate CA as the preferred scenario. This test was performed as described in de Oliveira Martins & Posada [30], where the columns of the alignment for one of the groups are randomly shuffled, such that the alignment is preserved within the group but disrupted between groups. After shuffling, the alignment is always optimized for the original dataset, so that the ML tree can be estimated and summary statistics can be calculated (see Material and methods). The results of the permutation test support the hypothesis of CA as the most likely scenario (electronic supplementary material, file S1). Nevertheless, this test also reveals that the second-best supported model—H1: IO for *E6* and *E7* in the Aves/Testudines clade—is not significantly worse. Thus, although the best-supported scenario is common origin for all extant *E6* and *E7* genes, we cannot reject the hypothesis that *E6* and *E7* in extant PVs infecting birds and turtles have originated independently from the *E6* and *E7* genes in extant PVs infecting mammals. Only a denser sampling of PV genomes from different large amniote clades outside mammals, as well as from monotremes and marsupials, may allow us to distinguish between these conflicting hypotheses.

**Table 2.** Testing for common ancestry of *E6* and *E7* on reduced dataset 1. The test was performed using the software Bali-Phy on the concatenated *E6* and *E7* amino acid sequences as well as on *E6* and *E7* separately. The log marginal likelihoods (P(data|M)) are indicated for the Common Ancestry (CA) model (H0) and the alternative Independent Origin (IO) models (H1–H7). The Bayes factor for CA is calculated as ΔBF = log [Prob(CA)] − log [Prob(IO)], such that positive values favour CA and negative values indicate IO.

	*E6E7*		*E6*		*E7*	
model	P(data|M)	ΔBF	P(data|M)	ΔBF	P(data|M)	ΔBF
H0: (grey-blue-yellow-red-black-green)	−19083.055	0	−10960.151	0	−8239.297	0
H1: grey+(blue-yellow-red-black-green)	−19425.259	342.204	−11008.514	48.363	−8300.605	61.308
H2: grey+blue+(yellow-red-black-green)	−19595.075	512.020	−11102.586	142.435	−8391.373	152.076
H3: grey+blue+yellow+(red-black-green)	−19883.098	800.043	−11266.682	306.531	−8515.244	275.947
H4: grey+blue+yellow+red+(black-green)	−20108.285	1025.230	−11390.535	430.384	−8644.525	405.228
H5: grey+blue+(red-black)+(green-yellow)	−19850.013	766.958	−11239.646	279.495	−8541.050	301.753
H6: grey+blue+red+black+(green-yellow)	−22355.489	3272.434	−11352.869	392.718	−8638.945	399.648
H7: grey+blue+yellow+red+black+green	−20317.714	1234.659	−11503.409	543.258	−8748.908	509.611

**Table 3.** Testing for common ancestry of *E6* and *E7* on reduced dataset 2. The test was performed using the software Bali-Phy on the concatenated *E6* and *E7* amino acid sequences as well as on *E6* and *E7* separately. The log marginal likelihoods (P(data|M)) are indicated for the Common Ancestry (CA) model (H0) and the alternative Independent Origin (IO) models (H1–H7). The Bayes factor for CA is calculated as ΔBF = log [Prob(CA)] − log [Prob(IO)], such that positive values favour CA and negative values indicate IO.

	*E6E7*		*E6*		*E7*	
model	P(data|M)	ΔBF	P(data|M)	ΔBF	P(data|M)	ΔBF
H0: (grey-blue-yellow-red-black-green)	−19083.055	0	−10847.336	0	−8106.947	0
H1: grey+(blue-yellow-red-black-green)	−19165.438	82.383	−10915.029	67.693	−8153.371	46.424
H2: grey+blue+(yellow-red-black-green)	−19406.063	323.008	−11020.832	173.496	−8281.371	174.424
H3: grey+blue+yellow+(red-black-green)	−19676.573	593.518	−11174.707	327.371	−8424.371	317.424
H4: grey+blue+yellow+red+(black-green)	−19921.623	838.568	−11306.744	459.408	−8539.261	432.314
H5: grey+blue+(red-black)+(green-yellow)	−19682.881	599.826	−13601.982	2754.646	−8411.536	304.589
H6: grey+blue+red+black+(green-yellow)	−19886.211	803.156	−13704.295	2856.959	−8511.299	404.352
H7: grey+blue+yellow+red+black+green	−20121.000	1037.945	−11412.956	565.620	−8642.184	535.237

### (d) The emergence of an oncogenic potential in certain human papillomaviruses

A well-substantiated body of scientific literature suggests that the oncogenic potential of certain HPVs lies in the perturbation of key cellular checkpoints by the E6 and E7 proteins. However, although most PVs contain these genes, only a few of them are actually associated with cancer. Among the more than 220 HPVs, around 20 closely related *AlphaPVs* have been classified by the International Agency for the Research on Cancer (IARC) as carcinogenic or potentially carcinogenic to humans. Apart from humans, most other cancer cases associated with PVs are rare: EcabPV2 in penile cancers in stallions [47], RaegPV1 in basosquamous carcinoma in the Egyptian fruit bat [40] or RrupPV1 in a nasolabial tumour in a free-raning chamois [48]. A more specific rare case is represented by bovine PVs (BPVs). These PVs induce benign tumours of cutaneous or mucosal epithelia in the cattle; however, in the case of BPV1 (and less common BPV2), a host switch reveals its oncogenic potential, as in horses BPV1 can give rise to malign fibroblastic tumours (sarcoids) [49]. The later classified BPV13 has also been found in equine sarcoids [50]. Nevertheless, BPV1 is also detected in the skin and blood of healthy horses [51,52], and one report suggests that equine-adapted BPV1 strains exist [52,53]. It has been proposed that BPV1 variant sequences are associated with either a benign or malign phenotype by altering the expression of the E5 protein [54]. Besides this host switch from a bovine PV to an equine host, eight different equine PVs have been described (PaVE: pave.niaid.nih.gov, [15]). *Equine papillomavirus 2* (EcabPV2) has been detected in genital squamous cell carcinomas (SCCs) and healthy genital mucosa [55,56]. However, odd ratios for the presence of viral material in diseased *versus* healthy animals indeed suggests that EcabPV2 contributes to the onset and progression of genital SCCs in horses [55,57]. Preliminary findings further suggest that EcabPV2 resides in infected cells as virions, viral episomes and integrated viral DNA [56], similar to cancer-associated HPVs [58]. This seems to be an example of convergent evolution, where EcabPV2 and oncogenic HPVs both evolved analogous mechanisms independently to stimulate the development of PV-associated cancers.

**Figure 2.**
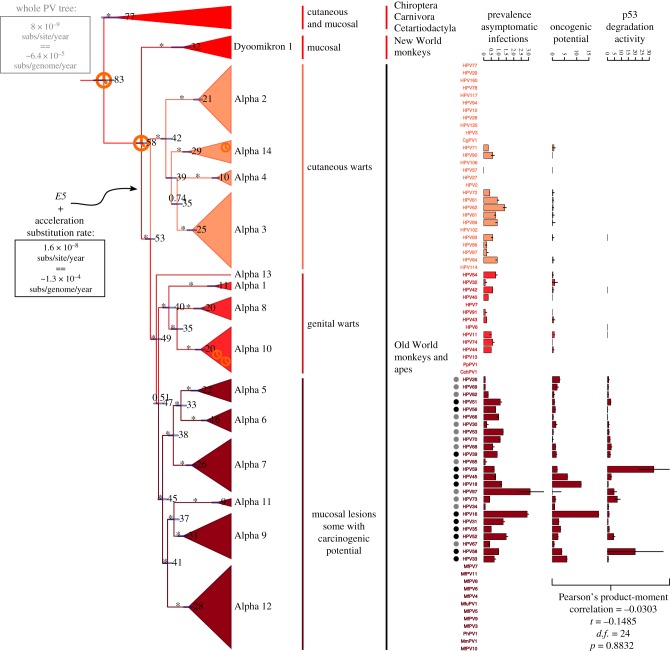
This tree is a zoom in on the Alpha–Omikron PV crown-group shown in figure 1. Values at the nodes correspond to posterior probabilities, where asterisks indicate full support. Error bars encompass 95% highest posterior density for the age of the nodes; next to the error bars, the median node age is given in millions of years ago (Ma). Clock symbols indicate the nodes used for calibration. A black arrow indicates the timing for the emergence of *E5* gene in the ancestral PV genome, between 53 and 58 Ma. Boxes display the average evolutionary rate for the complete PV tree (in grey) or for the *AlphaPV* subtree after the emergence of *E5* (in black). On the right side of the tree, the different PV species, the clinical presentation and host taxonomy are given. Dots label HPVs that have been classified by the IARC as carcinogenic to humans (black dots, group I) or probably/possibly carcinogenic to humans (grey dots, groups IIa and IIb). The three barplots on the right represent: (*a*) the worldwide prevalence of each HPV in women with normal cervical cytology, with error bars indicating the 95% confidence interval; (*b*) the oncogenic potential for each HPV, proxyed as the ratio between the prevalence of each HPV in cervical cancers divided by the prevalence in normal cervical cytology), with error bars indicating the 95% confidence interval; (*c*) the E6-mediated p53 degradation activity, expressed as the inverse value of the EC50 in ng of E6 protein needed to degrade cellular p53, with higher values indicating an enhanced potential of E6 to degrade p53; error bars indicate an approximate of the standard error of the mean. The first two barplots contain data obtained from the ICO/IARC HPV Information Centre (http://www.hpvcentre.net/), while the third contains data obtained from Mesplede *et al.* [7]. The correlation analysis for the second and third barplot is shown in the inset at the bottom. For the raw data of the barplots, see electronic supplementary material, table S4. (Online version in colour.)

Within the clade of PVs infecting primates (*AlphaPVs*: Catarrhini in red clade in figure 1), the E5 proteins are classified into different groups (E5*α*, E5*β*, E5*γ*, E5*δ*, E5*ε*, E5*ζ*) according to their hydrophobic profiles and phylogeny [3]. The *AlphaPVs* divide in three subclades with three different clinical presentations: cutaneous warts, genital warts and mucosal lesions (figure 2). The presence of a given E5 type strongly correlates with the clinical presentation of the corresponding PV infection: E5*α* is associated with malignant mucosal lesions, E5*β* is associated with benign cutaneous lesions, and the two putative proteins E5*γ* and E5*δ* are associated with benign mucosal lesions [3]. The appearance of the *E5* proto-oncogene in the ancestral *AlphaPV* genome can be dated back to between 53 and 58 Ma (figure 2), and concurred with an event that was instrumental for the differential oncogenic potential of present-day HPVs. One hypothesis is that the appearance of *E5* triggered an adaptive radiation that generated the three viral lineages with different clinical manifestations [1]. Nevertheless, we have recently shown that not all *E5*s have a common ancestor [43]. We interpret that *E5*s evolved *de novo* out of an initially non-coding region that integrated between the early and the late genes in the genome of an ancestral *AlphaPV* lineage. Interestingly, at the time of the integration of this non-coding region, we observe an acceleration of the evolutionary rate in the corresponding branch, two times higher than the overall PV substitution rate (figure 2). These results suggest the accommodation of the *E5*s in this region and the promotion of an adaptive radiation, where certain E6 (and probably also E7) proteins acquired the ability to degrade tumour suppressor proteins and facilitate the development of cancer in different tissues.

The oncogenic potential of HPVs strongly matches viral phylogeny [3,4]. The potential of p53 degradation by E6 proteins from *AlphaPVs* is highly correlated with such a phylogenetic grouping [6], which has suggested a mechanistic basis for the connection between phylogeny and oncogenicity. Although E6-mediated p53 degradation has always been considered one of the hallmarks of HPV-mediated cervical cancer [59], the connection between molecular mechanism and infection phenotype remains unclear. First, E6 proteins from non-oncogenic HPVs, notably HPV71, can also induce p53 degradation [6]. Second, rare albeit well-documented cases exist of malignancy associated with non-oncogenic HPVs, whose E6 proteins do not degrade p53 [60]. Finally, third, when the E6-mediated p53 degradation activity has been finely quantified [7], a more complex picture is revealed. Our thought-provoking results displayed in figure 2 clearly show that there is no correlation between the oncogenic potential for HPVs in cervical cancer, and the E6-mediated p53 degradation activity (Pearson’s product-moment correlation =−0.0303016, *t* = −0.14852, *d*.*f*. = 24, *p* = 0.8832). Cogent examples are HPV16 and HPV18, which display the highest oncogenic potential (proxyed as the ratio between worldwide prevalence in cervical cancers and worldwide prevalence in women with normal cervical cytology; data obtained from the ICO/IARC HPV Information Centre), but whose corresponding E6 proteins are not especially efficient at inducing p53 degradation (proxyed as the inverse of the EC50 concentration of E6 needed to degrade 50% of the cellular p53 protein, data obtained from [7]). For comparison, E6 from HPV58 requires 17-times less concentration to achieve the same p53 degradation effect as E6 from the closely related HPV16, and E6 from HPV59 requires 53-times less concentration to achieve as much p53 degradation as E6 from the closely related HPV18. Other factors, such as mRNA splicing and the presence of particular spliced E6 isoforms specific to oncogenic HPVs [61], may play essential roles in defining the overall oncogenic potential of the different HPVs.

## 4. Conclusion

In this study, we have revisited the evolution of PVs using phylogenetic dating on the largest and most diverse dataset compiled to date. The evolutionary scenario with the best explanatory power proposes an old ancestry for PVs, a primary radiation event that led to the generation of the different main lineages, a second radiation of the different lineages together with the expansion of their hosts, and a third radiation event specific to *AlphaPVs* after the emergence of the *E5* proto-oncogene. We identify further a number of anomalies in the PV tree that are inconsistent with this overall scenario. Some of these inconsistencies can be explained by lineage sorting and host switch events. We also show for the first time that the *E6* and *E7* oncogenes may have a common ancestor, although the alternative hypothesis of *E6* and *E7* from mammalian PVs and from Aves/Testudines PVs having independent origins cannot be rejected due to the still scarce sampling in these host clades. Overall, resolution of the deep nodes and fine support for the main scenario will require systematic sampling of PVs from other anamniotes, from amniotes other than mammals, from mammals other than placental mammals, and from placental mammals other than Laurasiatherians and Primates. We need to humbly admit that we are still far from understanding why PV-induced cervical cancer seems to be restricted to humans, as well as from identifying the molecular differences between closely related viruses underlying the enormous variance in the epidemiology of oncogenic PVs.

## Supplementary Material

Supplementary figures S1-S4

## Supplementary Material

Supplementary tables S1 to S4

## Supplementary Material

Supplementary file on permutation tests

## Data accessibility

Supplementary data and scripts are available at http://doi.org/10.5281/zenodo.2563206.
