# Supplementary material for: Origin and evolution of papillomavirus (onco)genes and genomes
Source: Philos Trans R Soc Lond B Biol Sci. 2019 Apr 8;374(1773):20180303. doi: 10.1098/rstb.2018.0303 (PMC6501903; doi:10.1098/rstb.2018.0303)

Figure S1: ML phylogenetic trees for a data set containing 343 PVs

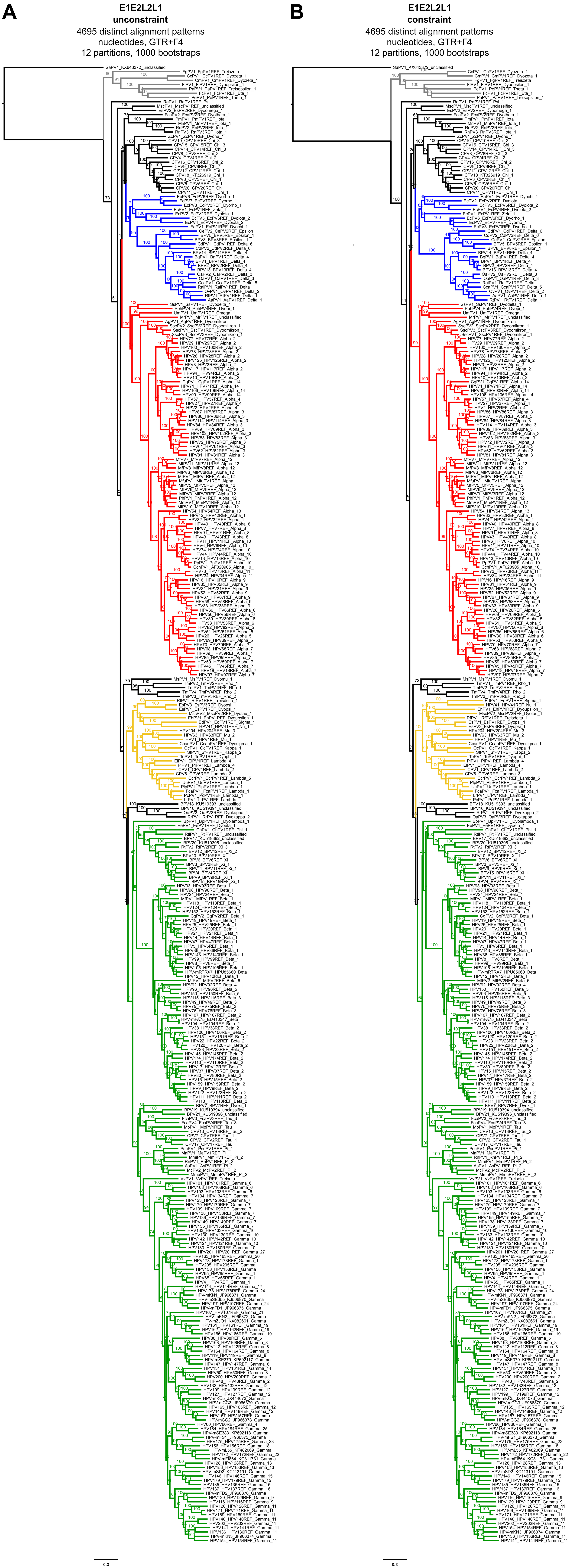

Figure S2: Dated Bayesian phylogenetic tree for a data set containing 343 PVs

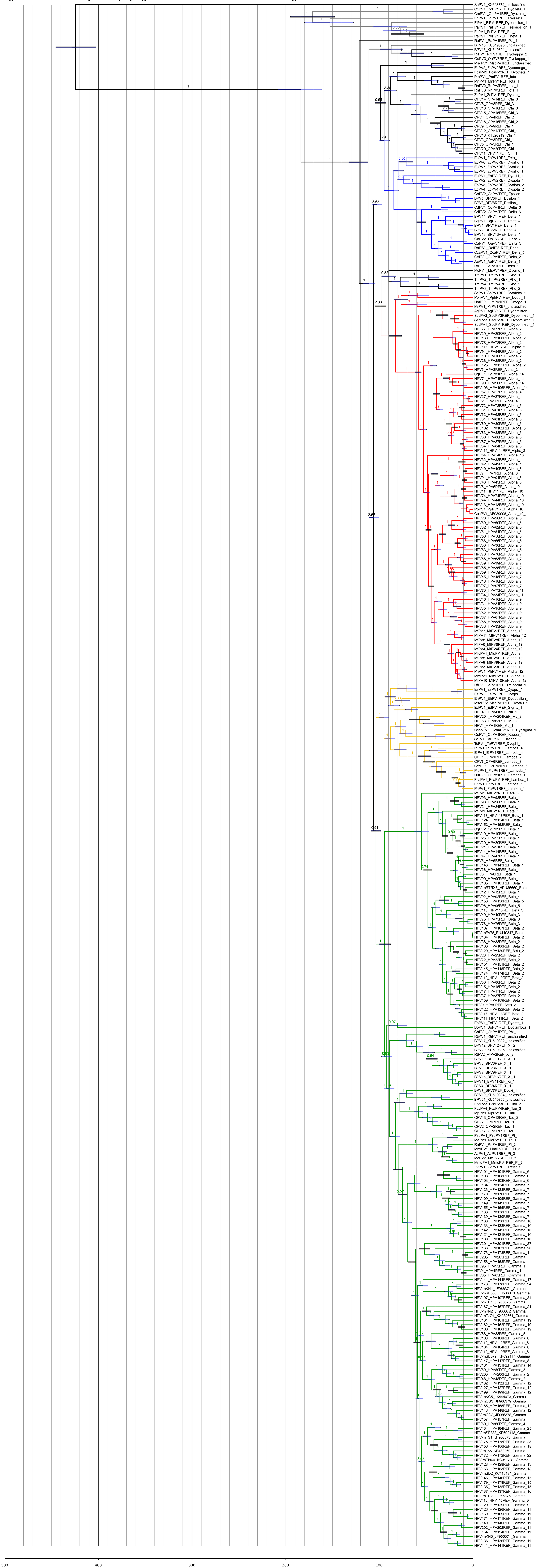

Figure S3: Posterior distribution of the inferred node age for the ancestors of the different PV clades

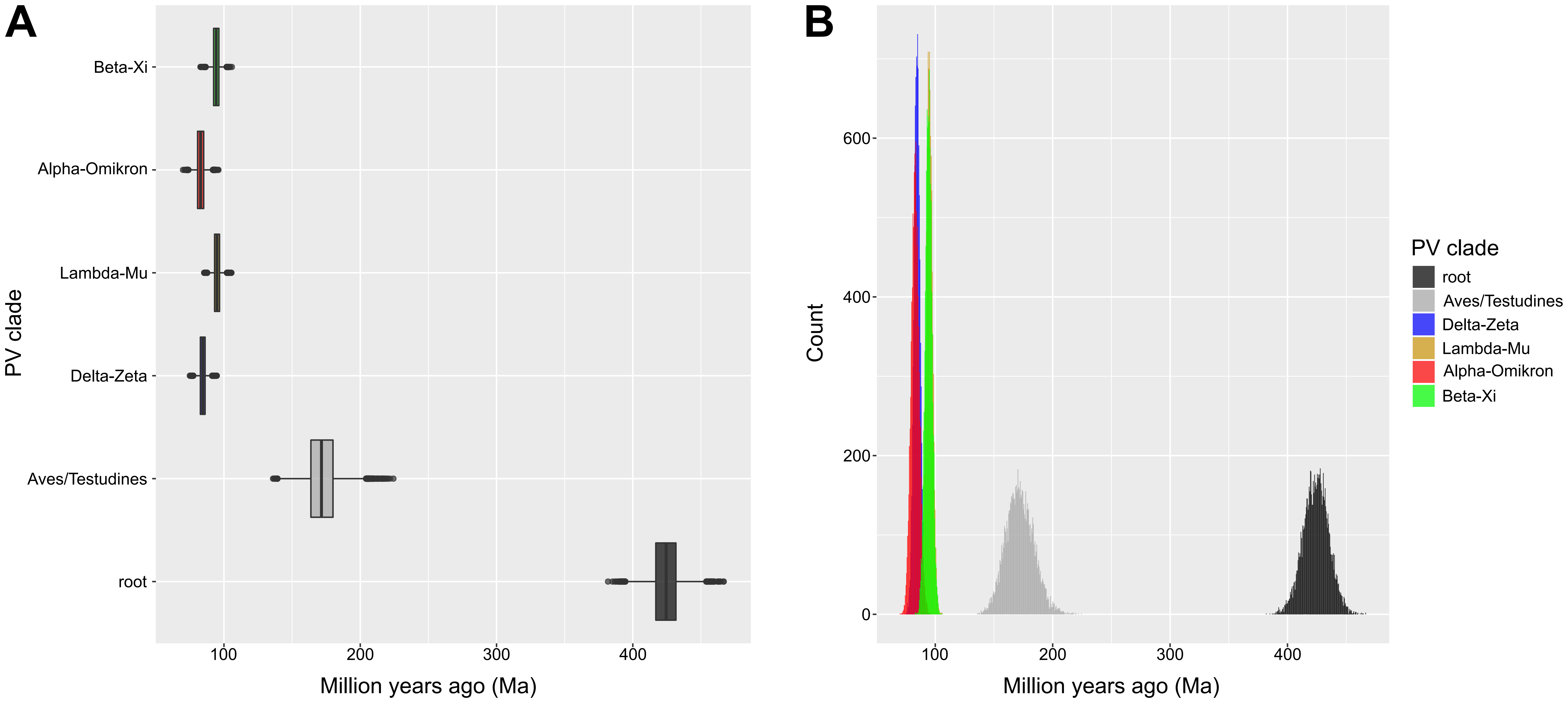

Figure S4: ML phylogenetic tree for a data set containing 348 PVs

E1E2L2L1

4912 distinct alignment patterns
nucleotides, GTR+Γ4
12 partitions, 1000 bootstraps

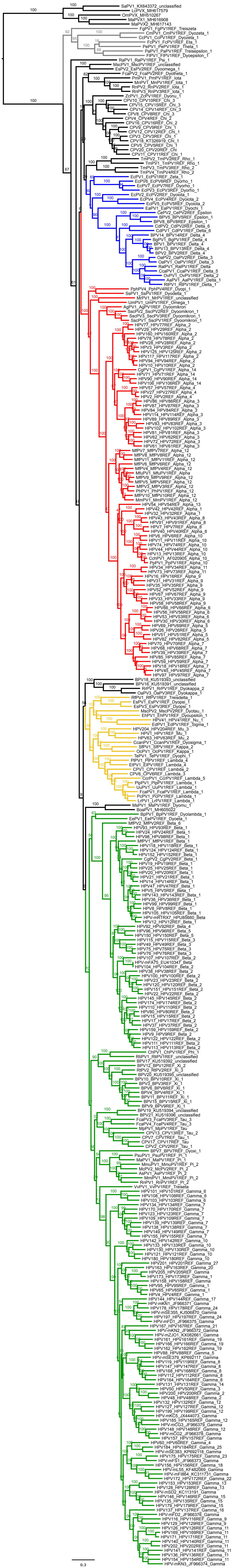

Supplement: Supplementary figures S1-S4 [file rstb20180303supp1.pdf]
