# Supplementary material for: Origin and evolution of papillomavirus (onco)genes and genomes
Source: Philos Trans R Soc Lond B Biol Sci. 2019 Apr 8;374(1773):20180303. doi: 10.1098/rstb.2018.0303 (PMC6501903; doi:10.1098/rstb.2018.0303)
Supplement: Supplementary file on permutation tests [file rstb20180303supp3.pdf]

Random permutations to test for common ancestry of E6 and E7.

To support the results of the Bali-Phy analyses, we performed a random permutation test as described in *de Oliveira Martins and Posada 2016*. In this test the sequences for one of the clades are randomly shuffled and statistics are recalculated after realignment with MUSCLE (*Edgar, 2004*), which tells us how much the original data depart from those with phylogenetic structure partially removed. The statistics used in this test are ML tree length (sum of the branch lengths) and Log likelihood, calculated with PhyMLv3.0 (*Guindon, 2010*).

We performed the random permutation test on the *E6* and *E7* PV oncogenes using two different reduced data sets (RD1 and RD2). Each data set contained 35 representative species from the different PV clades in fig. 1 , including the seven PVs infecting Aves and Testudines (grey clade), six Alpha-Omikron PVs (red clade), six Beta-Xi PVs (green clade), six Lambda-Mu PVs (yellow clade), six Delta-Zeta PVs (blue clade), and the four PVs infecting Manatees (black clade). The analyses were performed at the amino acid level using the LG substitution model, for E6 an E7 separately as well as concatenated. We assumed the null hypothesis (H0) of Common Ancestry (CA).

In our data set, we reshuffled one of the groups (in this case the Alpha-Omikron PV clade) 100 times, each time realigning against the other groups in the data set. For each iteration, the alignment is always optimised and the statistics (ML tree lengths and Log likelihoods) are calculated. To make the statistics comparable, the same alignment is used for both the Independent Origin (IO) and CA hypotheses. For the Log likelihoods, the Bayes Factor is then ΔBF= log[Prob(CA)]-log[Prob(IO)], such that positive values favor CA and negative values indicate IO.

The results are shown in the figures on the following pages, where on each page a different data set is depicted. The ML tree lengths are always shorter for H0, favoring CA for both *E6* and *E7*. The calculated Log likelihoods and ΔBF also indicate that CA is the best supported model. However, when comparing their distributions with a multiple comparison test after Kruskal-Wallis, the hypothesis H1: IO for *E6* and *E7* in the Aves/Testudines clade is not significantly worse. Thus, although the best scenario is common origin for all extant *E6* and *E7* genes, we cannot reject the hypothesis that *E6* and *E7* in extant PVs infecting birds and turtles have originated independently from the *E6* and *E7* genes in extant PVs infecting mammals.

Random Permutation Test

Reduced Dataset 1: E6E7

H0: (gray-blue-yellow-red-black-green)
H1: gray+(blue-yellow-red-black-green)
H2: gray+blue+(yellow-red-black-green)
H3: gray+blue+yellow+(red-black-green)
H4: gray+blue+yellow+red+(black-green)
H5: gray+blue+(red-black)+(green-yellow)
H6: gray+blue+red+black+(green-yellow)
H7: gray+blue+yellow+red+black+green

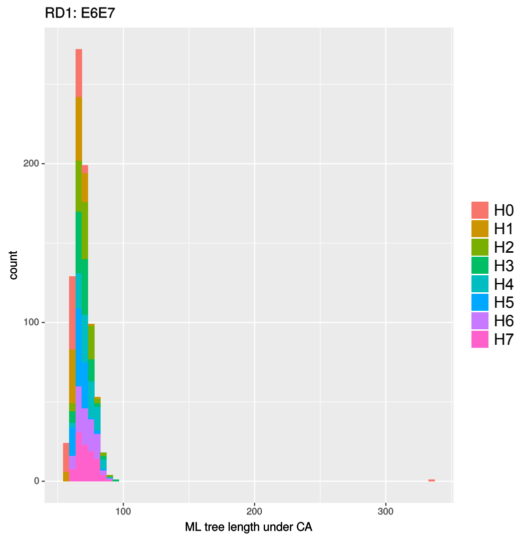

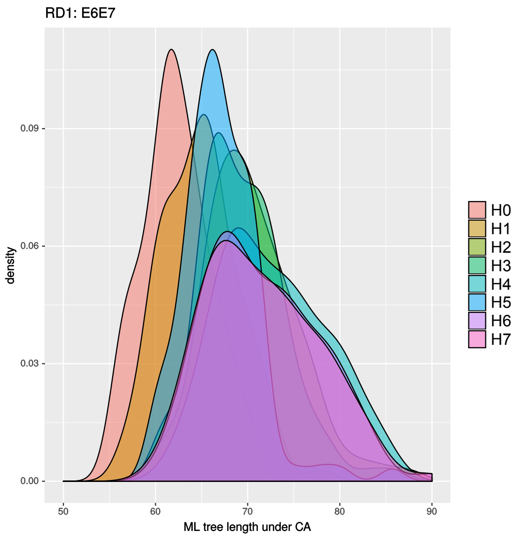

Kruskal-Wallis rank sum test on ML tree length
chi-squared = 276.65, df = 7, p-value < 2.2e-16

Multiple comparison test after Kruskal-Wallis
p.value: 0.05

	obs.dif	critical.dif	difference
tlen_H0-tlen_H1	93.73	102.0845	FALSE
tlen_H0-tlen_H2	330.76	102.0845	TRUE
tlen_H0-tlen_H3	296.09	102.0845	TRUE
tlen_H0-tlen_H4	405.87	102.0845	TRUE
tlen_H0-tlen_H5	187.34	102.0845	TRUE
tlen_H0-tlen_H6	361.87	102.0845	TRUE
tlen_H0-tlen_H7	357.54	102.0845	TRUE

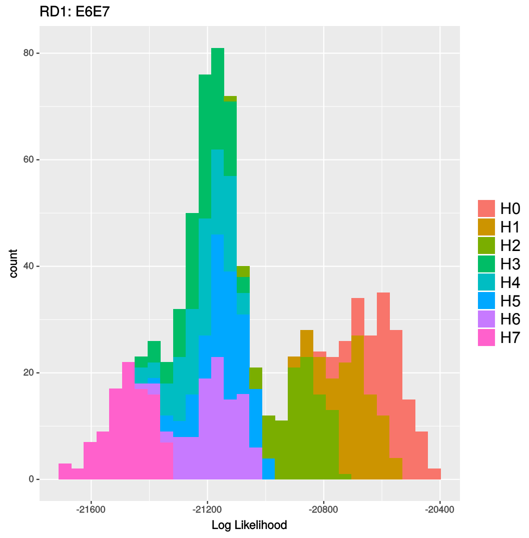

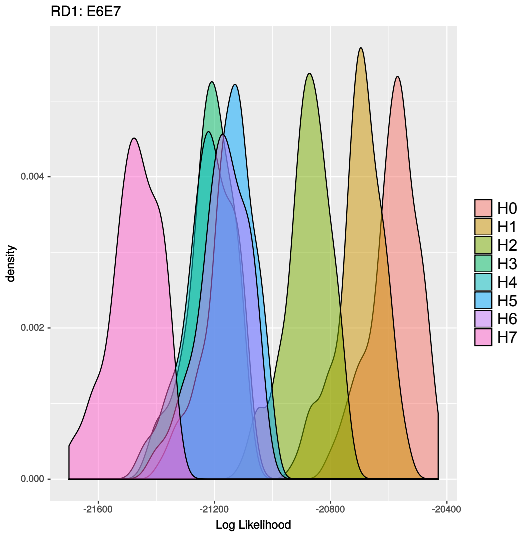

Kruskal-Wallis rank sum test on the Log Likelihood
chi-squared = 694.21, df = 7, p-value < 2.2e-16

Multiple comparison test after Kruskal-Wallis
p.value: 0.05

	obs.dif	critical.dif	difference
lnl_H0-lnl_H1	74.62	102.0845	FALSE
lnl_H0-lnl_H2	180.52	102.0845	TRUE
lnl_H0-lnl_H3	474.95	102.0845	TRUE
lnl_H0-lnl_H4	476.26	102.0845	TRUE
lnl_H0-lnl_H5	376.31	102.0845	TRUE
lnl_H0-lnl_H6	412.88	102.0845	TRUE
lnl_H0-lnl_H7	680.70	102.0845	TRUE

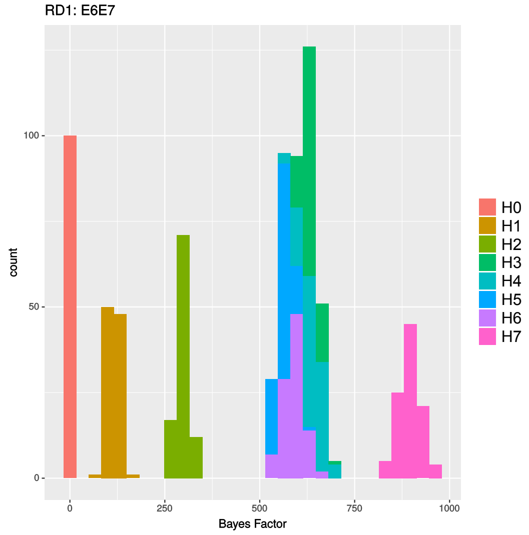

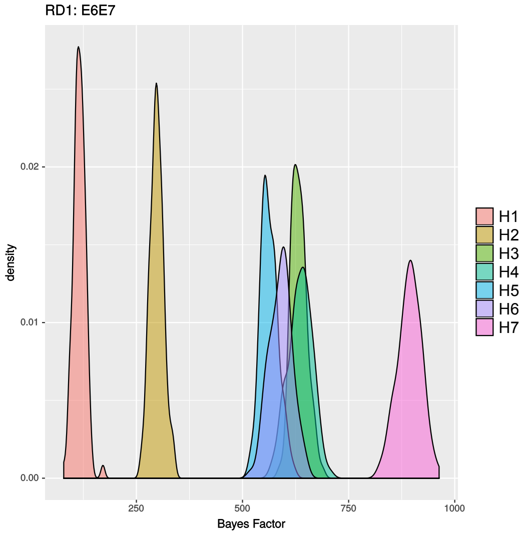

Kruskal-Wallis rank sum test on the ΔBF
chi-squared = 646.23, df = 6, p-value < 2.2e-16

Multiple comparison test after Kruskal-Wallis
p.value: 0.05

	obs.dif	critical.dif	difference
bf_H1-bf_H2	100.00	86.8822	TRUE
bf_H1-bf_H3	435.21	86.8822	TRUE
bf_H1-bf_H4	442.02	86.8822	TRUE
bf_H1-bf_H5	223.84	86.8822	TRUE
bf_H1-bf_H6	298.93	86.8822	TRUE
bf_H1-bf_H7	600.00	86.8822	TRUE
bf_H2-bf_H3	335.21	86.8822	TRUE
bf_H2-bf_H4	342.02	86.8822	TRUE
bf_H2-bf_H5	123.84	86.8822	TRUE
bf_H2-bf_H6	198.93	86.8822	TRUE
bf_H2-bf_H7	500.00	86.8822	TRUE
bf_H3-bf_H4	6.81	86.8822	FALSE
bf_H3-bf_H5	211.37	86.8822	TRUE
bf_H3-bf_H6	136.28	86.8822	TRUE
bf_H3-bf_H7	164.79	86.8822	TRUE
bf_H4-bf_H5	218.18	86.8822	TRUE
bf_H4-bf_H6	143.09	86.8822	TRUE
bf_H4-bf_H7	157.98	86.8822	TRUE
bf_H5-bf_H6	75.09	86.8822	FALSE
bf_H5-bf_H7	376.16	86.8822	TRUE
bf_H6-bf_H7	301.07	86.8822	TRUE

Random Permutation Test

Reduced Dataset 1: E6

H0: (gray-blue-yellow-red-black-green)
H1: gray+(blue-yellow-red-black-green)
H2: gray+blue+(yellow-red-black-green)
H3: gray+blue+yellow+(red-black-green)
H4: gray+blue+yellow+red+(black-green)
H5: gray+blue+(red-black)+(green-yellow)
H6: gray+blue+red+black+(green-yellow)
H7: gray+blue+yellow+red+black+green

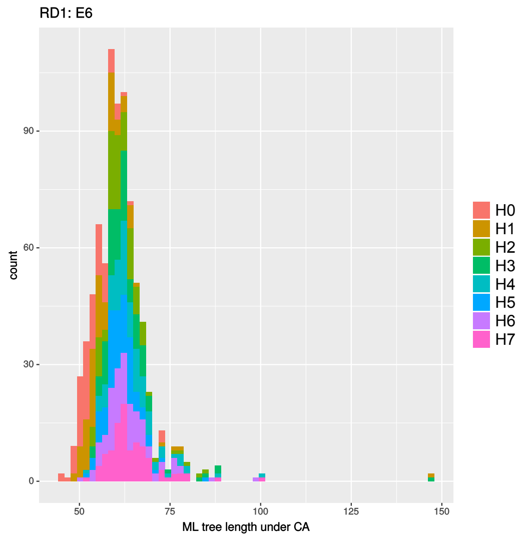

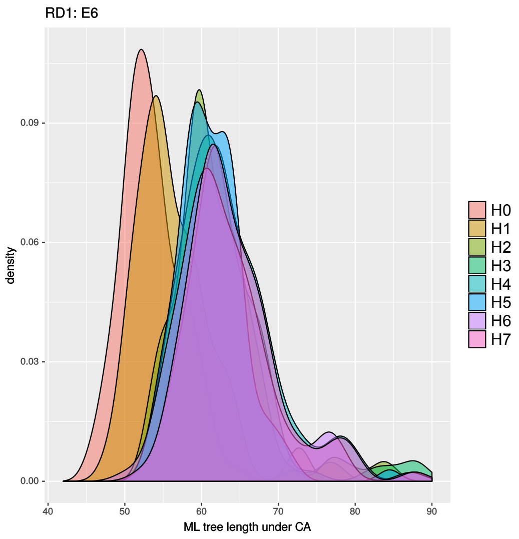

Kruskal-Wallis rank sum test on ML tree length
chi-squared = 267.71, df = 7, p-value < 2.2e-16

Multiple comparison test after Kruskal-Wallis
p.value: 0.05

	obs.dif	critical.dif	difference
tlen_H0-tlen_H1	81.97	102.0845	FALSE
tlen_H0-tlen_H2	293.32	102.0845	TRUE
tlen_H0-tlen_H3	322.51	102.0845	TRUE
tlen_H0-tlen_H4	390.28	102.0845	TRUE
tlen_H0-tlen_H5	286.30	102.0845	TRUE
tlen_H0-tlen_H6	341.05	102.0845	TRUE
tlen_H0-tlen_H7	380.17	102.0845	TRUE

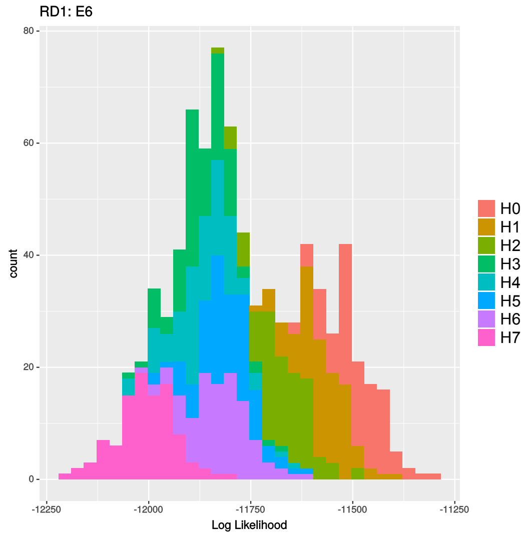

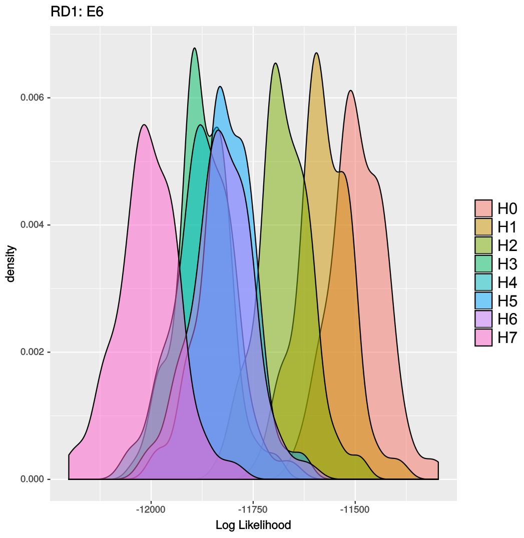

Kruskal-Wallis rank sum test on the Log Likelihood
chi-squared = 657.55, df = 7, p-value < 2.2e-16

Multiple comparison test after Kruskal-Wallis
p.value: 0.05

	obs.dif	critical.dif	difference
lnl_H0-lnl_H1	77.79	102.0845	FALSE
lnl_H0-lnl_H2	179.79	102.0845	TRUE
lnl_H0-lnl_H3	483.00	102.0845	TRUE
lnl_H0-lnl_H4	473.91	102.0845	TRUE
lnl_H0-lnl_H5	373.19	102.0845	TRUE
lnl_H0-lnl_H6	396.07	102.0845	TRUE
lnl_H0-lnl_H7	655.13	102.0845	TRUE

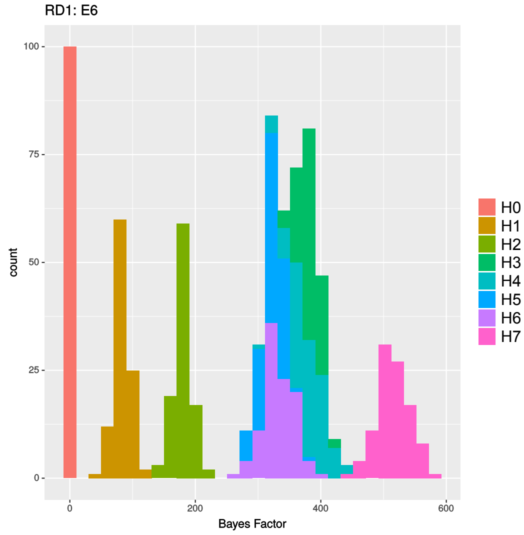

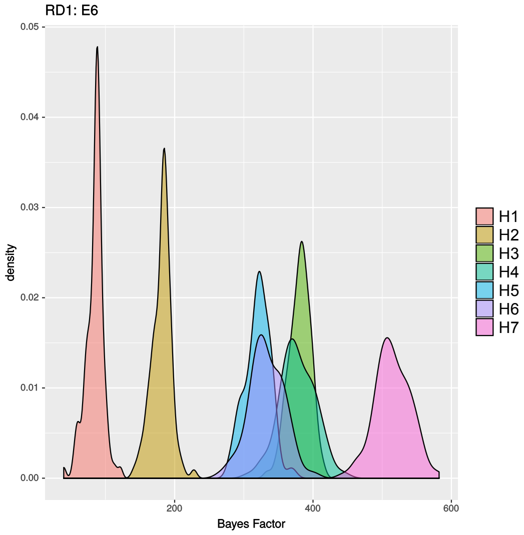

Kruskal-Wallis rank sum test on the ΔBF
chi-squared = 644.87, df = 6, p-value < 2.2e-16

Multiple comparison test after Kruskal-Wallis
p.value: 0.05

	obs.dif	critical.dif	difference
bf_H1-bf_H2	100.00	86.8822	TRUE
bf_H1-bf_H3	449.43	86.8822	TRUE
bf_H1-bf_H4	431.25	86.8822	TRUE
bf_H1-bf_H5	238.54	86.8822	TRUE
bf_H1-bf_H6	280.79	86.8822	TRUE
bf_H1-bf_H7	599.99	86.8822	TRUE
bf_H2-bf_H3	349.43	86.8822	TRUE
bf_H2-bf_H4	331.25	86.8822	TRUE
bf_H2-bf_H5	138.54	86.8822	TRUE
bf_H2-bf_H6	180.79	86.8822	TRUE
bf_H2-bf_H7	499.99	86.8822	TRUE
bf_H3-bf_H4	18.18	86.8822	FALSE
bf_H3-bf_H5	210.89	86.8822	TRUE
bf_H3-bf_H6	168.64	86.8822	TRUE
bf_H3-bf_H7	150.56	86.8822	TRUE
bf_H4-bf_H5	192.71	86.8822	TRUE
bf_H4-bf_H6	150.46	86.8822	TRUE
bf_H4-bf_H7	168.74	86.8822	TRUE
bf_H5-bf_H6	42.25	86.8822	FALSE
bf_H5-bf_H7	361.45	86.8822	TRUE
bf_H6-bf_H7	319.20	86.8822	TRUE

Random Permutation Test

Reduced Dataset 1: E7

H0: (gray-blue-yellow-red-black-green)
H1: gray+(blue-yellow-red-black-green)
H2: gray+blue+(yellow-red-black-green)
H3: gray+blue+yellow+(red-black-green)
H4: gray+blue+yellow+red+(black-green)
H5: gray+blue+(red-black)+(green-yellow)
H6: gray+blue+red+black+(green-yellow)
H7: gray+blue+yellow+red+black+green

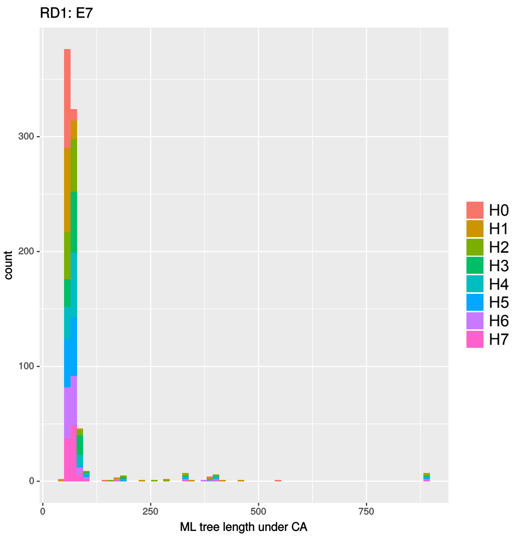

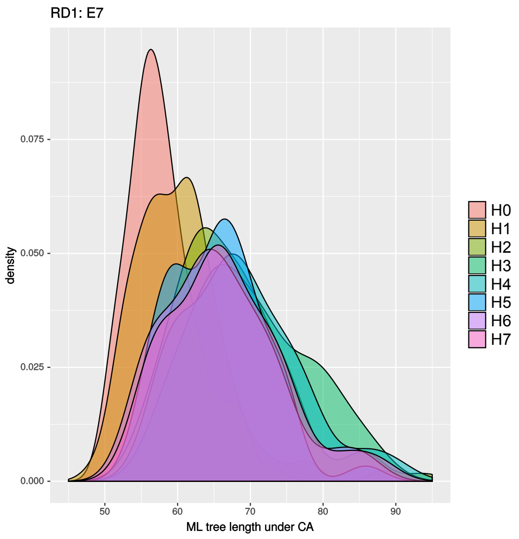

Kruskal-Wallis rank sum test on ML tree length
chi-squared = 165.86, df = 7, p-value < 2.2e-16

Multiple comparison test after Kruskal-Wallis
p.value: 0.05

	obs.dif	critical.dif	difference
tlen_H0-tlen_H1	97.52	102.0845	FALSE
tlen_H0-tlen_H2	269.31	102.0845	TRUE
tlen_H0-tlen_H3	335.53	102.0845	TRUE
tlen_H0-tlen_H4	308.82	102.0845	TRUE
tlen_H0-tlen_H5	227.26	102.0845	TRUE
tlen_H0-tlen_H6	234.92	102.0845	TRUE
tlen_H0-tlen_H7	256.56	102.0845	TRUE

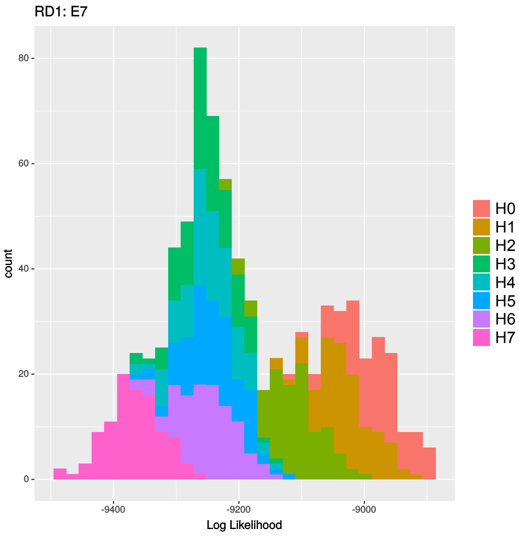

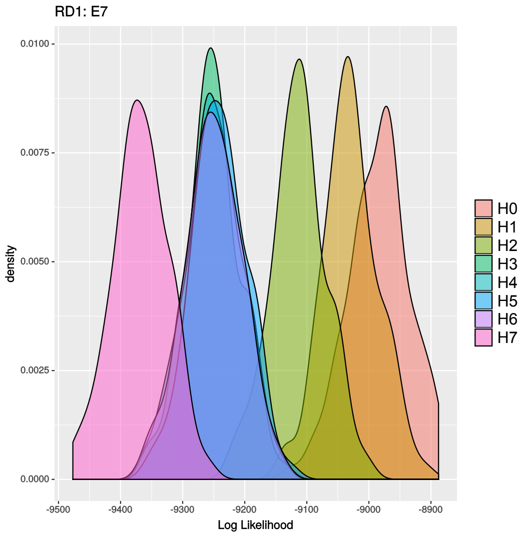

Kruskal-Wallis rank sum test on the Log Likelihood
chi-squared = 665.11, df = 7, p-value < 2.2e-16

Multiple comparison test after Kruskal-Wallis
p.value: 0.05

	obs.dif	critical.dif	difference
lnl_H0-lnl_H1	57.17	102.0845	FALSE
lnl_H0-lnl_H2	167.52	102.0845	TRUE
lnl_H0-lnl_H3	431.76	102.0845	TRUE
lnl_H0-lnl_H4	429.51	102.0845	TRUE
lnl_H0-lnl_H5	406.16	102.0845	TRUE
lnl_H0-lnl_H6	428.60	102.0845	TRUE
lnl_H0-lnl_H7	661.76	102.0845	TRUE

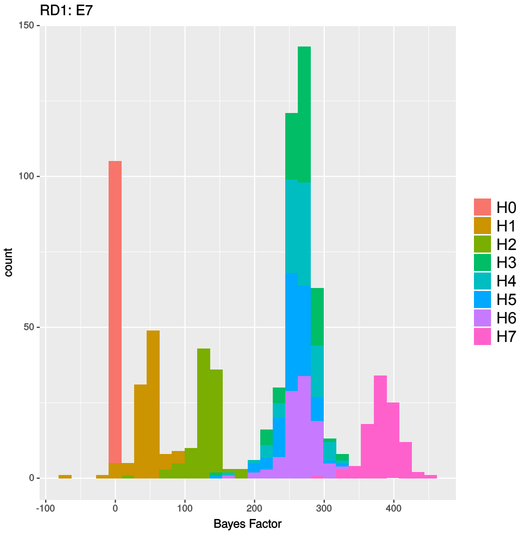

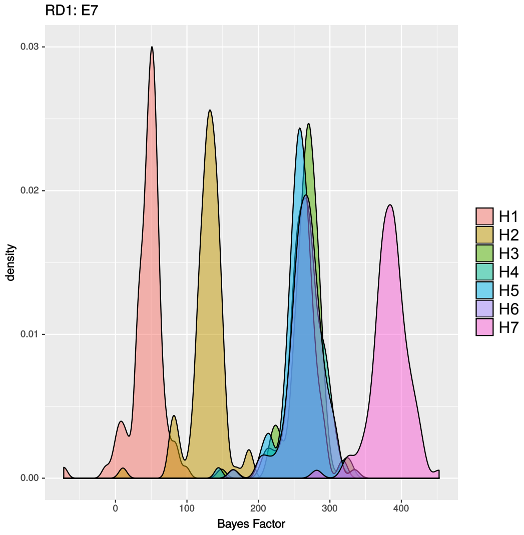

Kruskal-Wallis rank sum test on the ΔBF
chi-squared = 565.76, df = 6, p-value < 2.2e-16

Multiple comparison test after Kruskal-Wallis
p.value: 0.05

	obs.dif	critical.dif	difference
bf_H1-bf_H2	98.05	86.8822	TRUE
bf_H1-bf_H3	368.34	86.8822	TRUE
bf_H1-bf_H4	358.75	86.8822	TRUE
bf_H1-bf_H5	309.04	86.8822	TRUE
bf_H1-bf_H6	359.78	86.8822	TRUE
bf_H1-bf_H7	597.99	86.8822	TRUE
bf_H2-bf_H3	270.29	86.8822	TRUE
bf_H2-bf_H4	260.70	86.8822	TRUE
bf_H2-bf_H5	210.99	86.8822	TRUE
bf_H2-bf_H6	261.73	86.8822	TRUE
bf_H2-bf_H7	499.94	86.8822	TRUE
bf_H3-bf_H4	9.59	86.8822	FALSE
bf_H3-bf_H5	59.30	86.8822	FALSE
bf_H3-bf_H6	8.56	86.8822	FALSE
bf_H3-bf_H7	229.65	86.8822	TRUE
bf_H4-bf_H5	49.71	86.8822	FALSE
bf_H4-bf_H6	1.03	86.8822	FALSE
bf_H4-bf_H7	239.24	86.8822	TRUE
bf_H5-bf_H6	50.74	86.8822	FALSE
bf_H5-bf_H7	288.95	86.8822	TRUE
bf_H6-bf_H7	238.21	86.8822	TRUE

Random Permutation Test

Reduced Dataset 2: E6E7

H0: (gray-blue-yellow-red-black-green)
H1: gray+(blue-yellow-red-black-green)
H2: gray+blue+(yellow-red-black-green)
H3: gray+blue+yellow+(red-black-green)
H4: gray+blue+yellow+red+(black-green)
H5: gray+blue+(red-black)+(green-yellow)
H6: gray+blue+red+black+(green-yellow)
H7: gray+blue+yellow+red+black+green

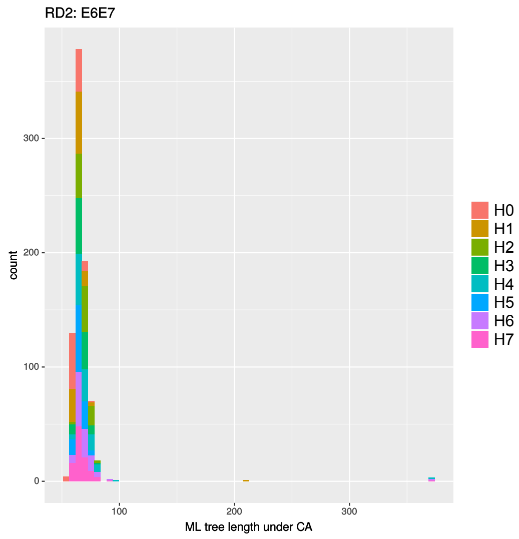

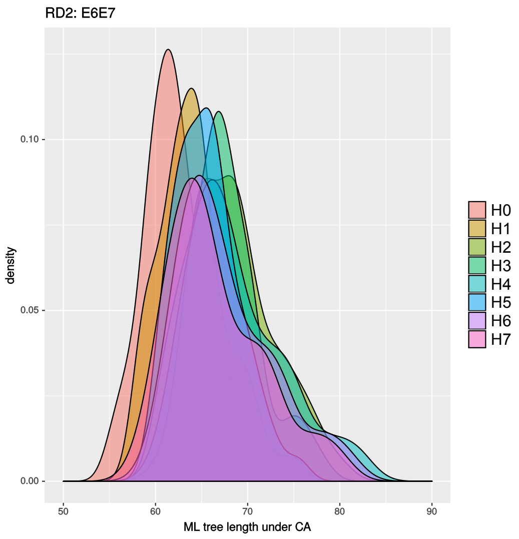

Kruskal-Wallis rank sum test on ML tree length
chi-squared = 167.42, df = 7, p-value < 2.2e-16

Multiple comparison test after Kruskal-Wallis
p.value: 0.05

	obs.dif	critical.dif	difference
tlen_H0-tlen_H1	101.57	102.0845	FALSE
tlen_H0-tlen_H2	328.90	102.0845	TRUE
tlen_H0-tlen_H3	263.70	102.0845	TRUE
tlen_H0-tlen_H4	320.92	102.0845	TRUE
tlen_H0-tlen_H5	180.01	102.0845	TRUE
tlen_H0-tlen_H6	267.30	102.0845	TRUE
tlen_H0-tlen_H7	216.08	102.0845	TRUE

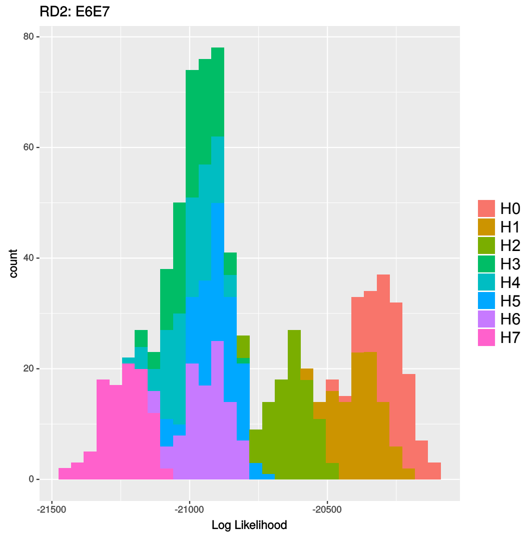

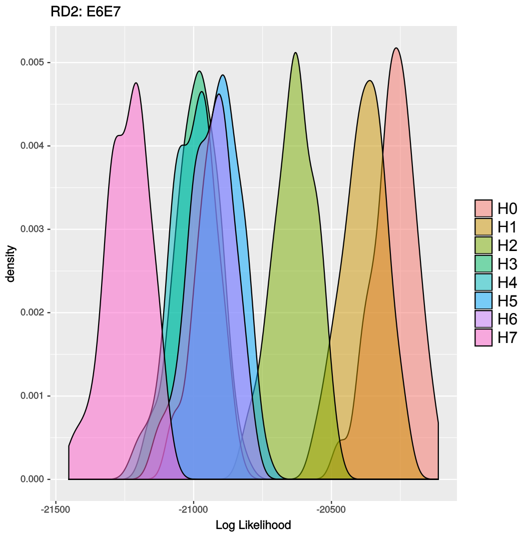

Kruskal-Wallis rank sum test on the Log Likelihood
chi-squared = 705.72, df = 7, p-value < 2.2e-16

Multiple comparison test after Kruskal-Wallis
p.value: 0.05

	obs.dif	critical.dif	difference
llnl_H0-lnl_H1	68.72	102.0845	FALSE
lnl_H0-lnl_H2	183.82	102.0845	TRUE
lnl_H0-lnl_H3	475.45	102.0845	TRUE
lnl_H0-lnl_H4	494.20	102.0845	TRUE
lnl_H0-lnl_H5	360.52	102.0845	TRUE
lnl_H0-lnl_H6	407.66	102.0845	TRUE
lnl_H0-lnl_H7	681.23	102.0845	TRUE

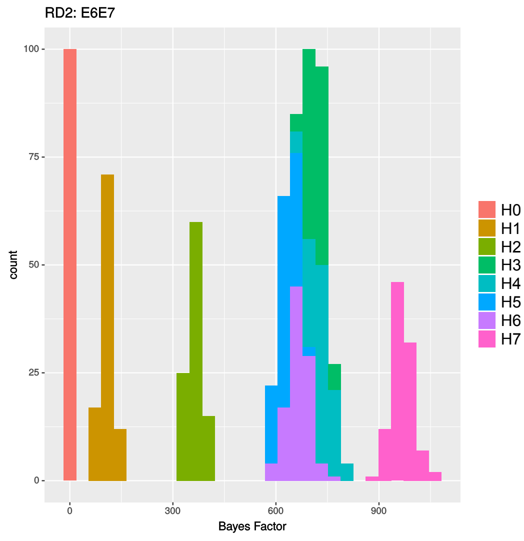

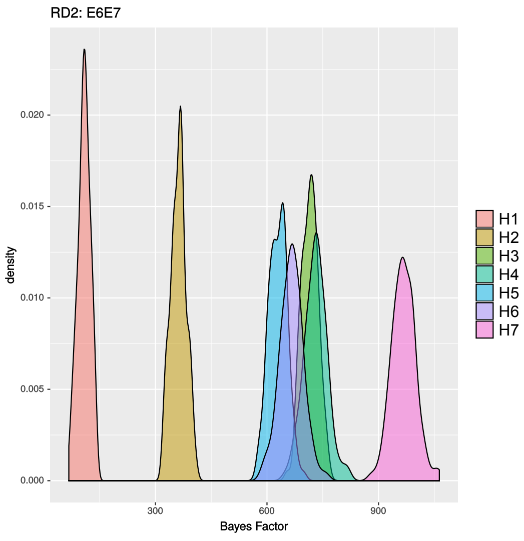

Kruskal-Wallis rank sum test on the ΔBF
chi-squared = 654.07, df = 6, p-value < 2.2e-16

Multiple comparison test after Kruskal-Wallis
p.value: 0.05

	obs.dif	critical.dif	difference
bf_H1-bf_H2	100.00	86.8822	TRUE
bf_H1-bf_H3	423.75	86.8822	TRUE
bf_H1-bf_H4	459.52	86.8822	TRUE
bf_H1-bf_H5	218.17	86.8822	TRUE
bf_H1-bf_H6	298.56	86.8822	TRUE
bf_H1-bf_H7	600.00	86.8822	TRUE
bf_H2-bf_H3	323.75	86.8822	TRUE
bf_H2-bf_H4	359.52	86.8822	TRUE
bf_H2-bf_H5	118.17	86.8822	TRUE
bf_H2-bf_H6	198.56	86.8822	TRUE
bf_H2-bf_H7	500.00	86.8822	TRUE
bf_H3-bf_H4	35.77	86.8822	FALSE
bf_H3-bf_H5	205.58	86.8822	TRUE
bf_H3-bf_H6	125.19	86.8822	TRUE
bf_H3-bf_H7	176.25	86.8822	TRUE
bf_H4-bf_H5	241.35	86.8822	TRUE
bf_H4-bf_H6	160.96	86.8822	TRUE
bf_H4-bf_H7	140.48	86.8822	TRUE
bf_H5-bf_H6	80.39	86.8822	FALSE
bf_H5-bf_H7	381.83	86.8822	TRUE
bf_H6-bf_H7	301.44	86.8822	TRUE

Random Permutation Test

Reduced Dataset 2: E6

H0: (gray-blue-yellow-red-black-green)
H1: gray+(blue-yellow-red-black-green)
H2: gray+blue+(yellow-red-black-green)
H3: gray+blue+yellow+(red-black-green)
H4: gray+blue+yellow+red+(black-green)
H5: gray+blue+(red-black)+(green-yellow)
H6: gray+blue+red+black+(green-yellow)
H7: gray+blue+yellow+red+black+green

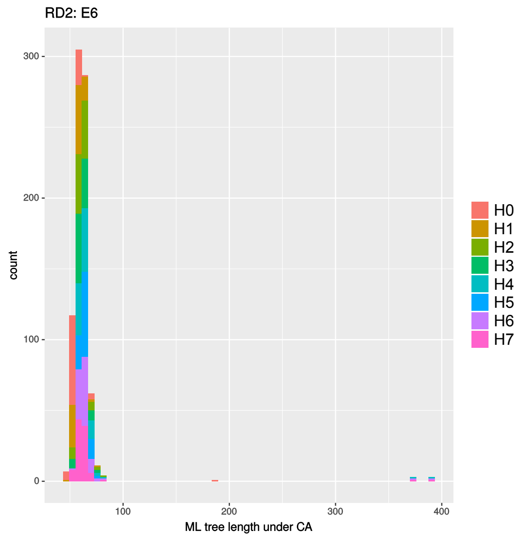

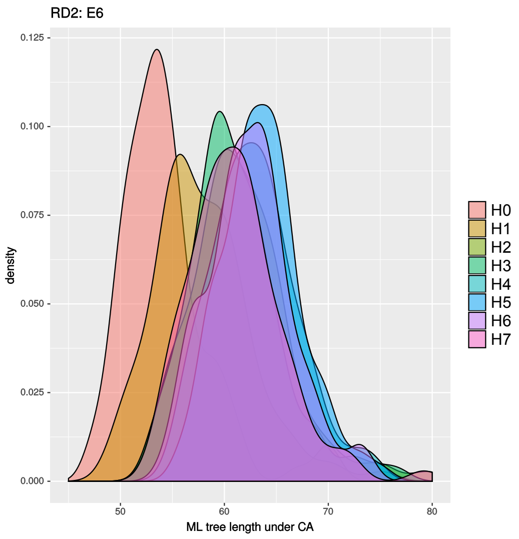

Kruskal-Wallis rank sum test on ML tree length
chi-squared = 245.99, df = 7, p-value < 2.2e-16

Multiple comparison test after Kruskal-Wallis
p.value: 0.05

	obs.dif	critical.dif	difference
tlen_H0-tlen_H1	127.82	102.0845	TRUE
tlen_H0-tlen_H2	298.49	102.0845	TRUE
tlen_H0-tlen_H3	272.81	102.0845	TRUE
tlen_H0-tlen_H4	376.16	102.0845	TRUE
tlen_H0-tlen_H5	404.37	102.0845	TRUE
tlen_H0-tlen_H6	359.76	102.0845	TRUE
tlen_H0-tlen_H7	284.35	102.0845	TRUE

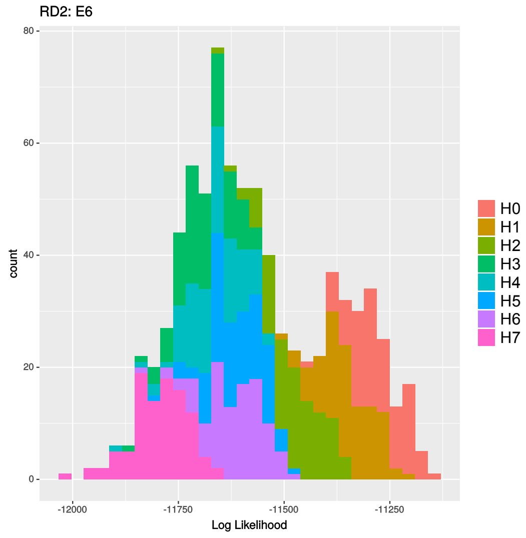

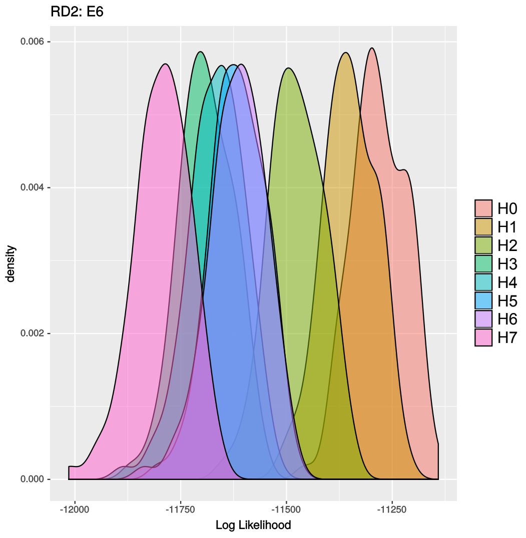

Kruskal-Wallis rank sum test on the Log Likelihood
chi-squared = 672.47, df = 7, p-value < 2.2e-16

Multiple comparison test after Kruskal-Wallis
p.value: 0.05

	obs.dif	critical.dif	difference
lnl_H0-lnl_H1	62.68	102.0845	FALSE
lnl_H0-lnl_H2	181.64	102.0845	TRUE
lnl_H0-lnl_H3	508.34	102.0845	TRUE
lnl_H0-lnl_H4	464.06	102.0845	TRUE
lnl_H0-lnl_H5	373.04	102.0845	TRUE
lnl_H0-lnl_H6	373.03	102.0845	TRUE
lnl_H0-lnl_H7	651.45	102.0845	TRUE

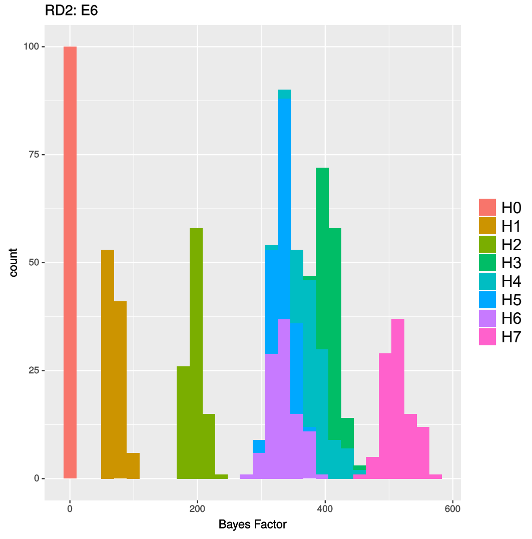

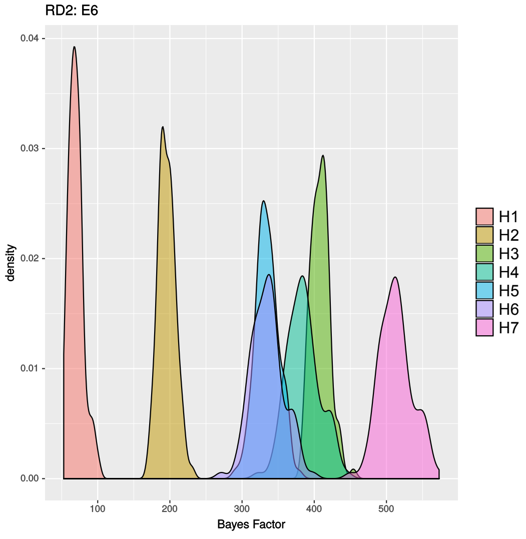

Kruskal-Wallis rank sum test on the ΔBF
chi-squared = 658.24, df = 6, p-value < 2.2e-16

Multiple comparison test after Kruskal-Wallis
p.value: 0.05

	obs.dif	critical.dif	difference
bf_H1-bf_H2	100.00	86.8822	TRUE
bf_H1-bf_H3	481.73	86.8822	TRUE
bf_H1-bf_H4	409.48	86.8822	TRUE
bf_H1-bf_H5	253.42	86.8822	TRUE
bf_H1-bf_H6	255.38	86.8822	TRUE
bf_H1-bf_H7	599.99	86.8822	TRUE
bf_H2-bf_H3	381.73	86.8822	TRUE
bf_H2-bf_H4	309.48	86.8822	TRUE
bf_H2-bf_H5	153.42	86.8822	TRUE
bf_H2-bf_H6	155.38	86.8822	TRUE
bf_H2-bf_H7	499.99	86.8822	TRUE
bf_H3-bf_H4	72.25	86.8822	FALSE
bf_H3-bf_H5	228.31	86.8822	TRUE
bf_H3-bf_H6	226.35	86.8822	TRUE
bf_H3-bf_H7	118.26	86.8822	TRUE
bf_H4-bf_H5	156.06	86.8822	TRUE
bf_H4-bf_H6	154.10	86.8822	TRUE
bf_H4-bf_H7	190.51	86.8822	TRUE
bf_H5-bf_H6	1.96	86.8822	FALSE
bf_H5-bf_H7	346.57	86.8822	TRUE
bf_H6-bf_H7	344.61	86.8822	TRUE

Random Permutation Test

Reduced Dataset 2: E7

H0: (gray-blue-yellow-red-black-green)
H1: gray+(blue-yellow-red-black-green)
H2: gray+blue+(yellow-red-black-green)
H3: gray+blue+yellow+(red-black-green)
H4: gray+blue+yellow+red+(black-green)
H5: gray+blue+(red-black)+(green-yellow)
H6: gray+blue+red+black+(green-yellow)
H7: gray+blue+yellow+red+black+green

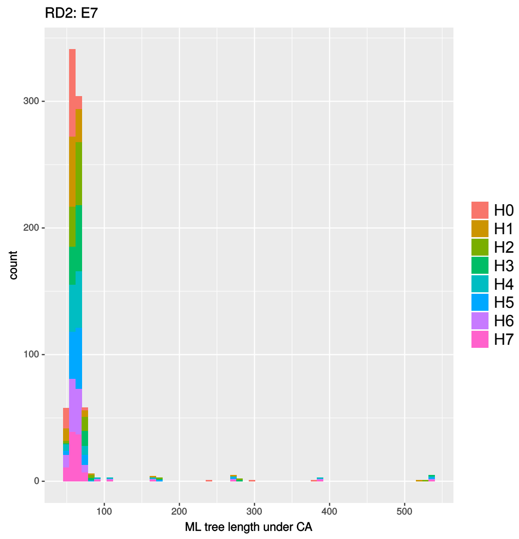

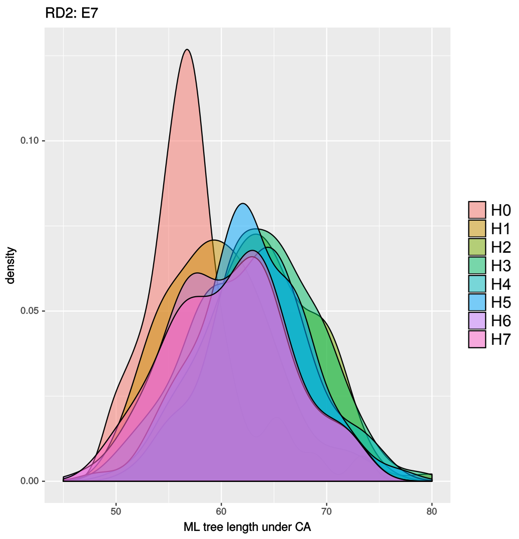

Kruskal-Wallis rank sum test on ML tree length
chi-squared = 110.41, df = 7, p-value < 2.2e-16

Multiple comparison test after Kruskal-Wallis
p.value: 0.05

	obs.dif	critical.dif	difference
tlen_H0-tlen_H1	100.82	102.0845	FALSE
tlen_H0-tlen_H2	258.38	102.0845	TRUE
tlen_H0-tlen_H3	278.32	102.0845	TRUE
tlen_H0-tlen_H4	220.37	102.0845	TRUE
tlen_H0-tlen_H5	224.17	102.0845	TRUE
tlen_H0-tlen_H6	154.42	102.0845	TRUE
tlen_H0-tlen_H7	157.36	102.0845	TRUE

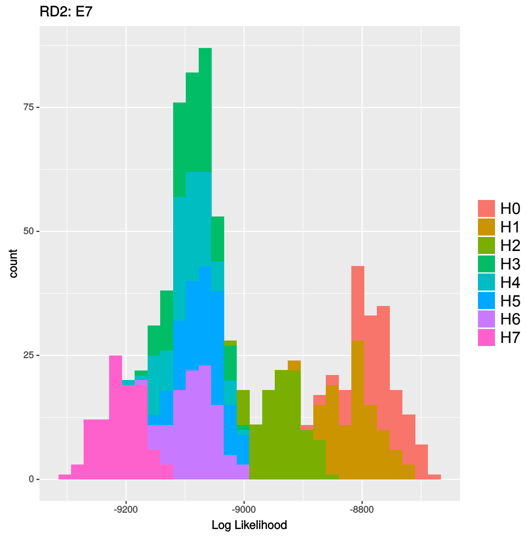

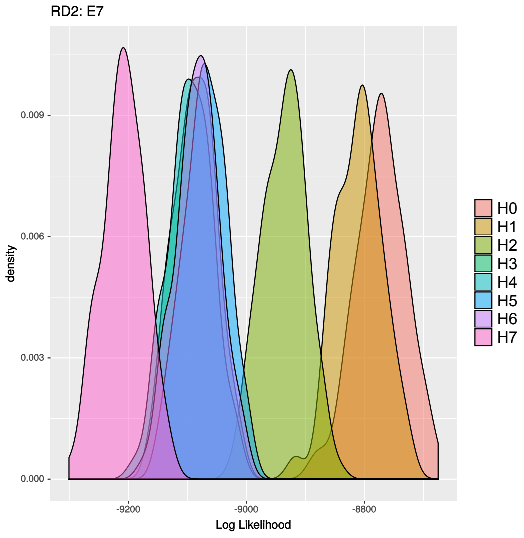

Kruskal-Wallis rank sum test on the Log Likelihood
chi-squared = 688.43, df = 7, p-value < 2.2e-16

Multiple comparison test after Kruskal-Wallis
p.value: 0.05

	obs.dif	critical.dif	difference
lnl_H0-lnl_H1	49.38	102.0845	FALSE
lnl_H0-lnl_H2	173.36	102.0845	TRUE
lnl_H0-lnl_H3	436.45	102.0845	TRUE
lnl_H0-lnl_H4	459.10	102.0845	TRUE
lnl_H0-lnl_H5	381.42	102.0845	TRUE
lnl_H0-lnl_H6	421.58	102.0845	TRUE
lnl_H0-lnl_H7	671.59	102.0845	TRUE

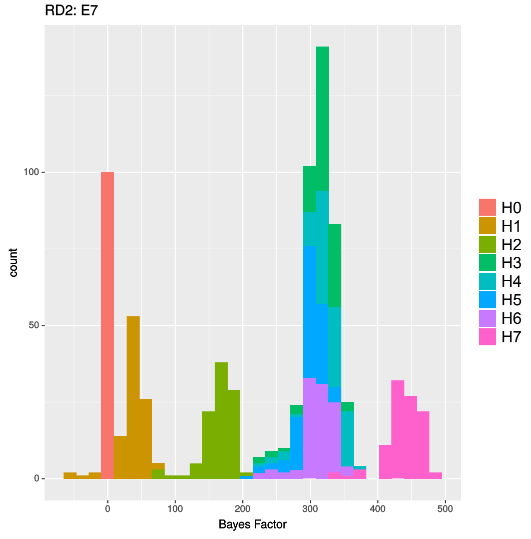

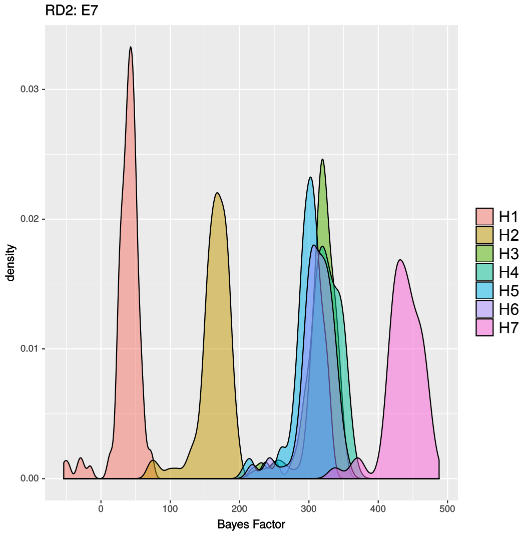

Kruskal-Wallis rank sum test on the ΔBF
chi-squared = 565.76, df = 6, p-value < 2.2e-16

Multiple comparison test after Kruskal-Wallis
p.value: 0.05

	obs.dif	critical.dif	difference
bf_H1-bf_H2	99.96	86.8822	TRUE
bf_H1-bf_H3	373.27	86.8822	TRUE
bf_H1-bf_H4	410.86	86.8822	TRUE
bf_H1-bf_H5	271.96	86.8822	TRUE
bf_H1-bf_H6	344.92	86.8822	TRUE
bf_H1-bf_H7	598.89	86.8822	TRUE
bf_H2-bf_H3	273.31	86.8822	TRUE
bf_H2-bf_H4	310.90	86.8822	TRUE
bf_H2-bf_H5	172.00	86.8822	TRUE
bf_H2-bf_H6	244.96	86.8822	TRUE
bf_H2-bf_H7	498.93	86.8822	TRUE
bf_H3-bf_H4	37.59	86.8822	FALSE
bf_H3-bf_H5	101.31	86.8822	TRUE
bf_H3-bf_H6	28.35	86.8822	FALSE
bf_H3-bf_H7	225.62	86.8822	TRUE
bf_H4-bf_H5	138.90	86.8822	TRUE
bf_H4-bf_H6	65.94	86.8822	FALSE
bf_H4-bf_H7	188.03	86.8822	TRUE
bf_H5-bf_H6	72.96	86.8822	FALSE
bf_H5-bf_H7	326.93	86.8822	TRUE
bf_H6-bf_H7	253.97	86.8822	TRUE
